# Dissecting the Spatial and Single‐Cell Transcriptomic Architecture of Cancer Stem Cell Niche Driving Tumor Progression in Gastric Cancer

**DOI:** 10.1002/advs.202413019

**Published:** 2025-02-14

**Authors:** Guangyu Zhang, Xin Zhang, Wenting Pan, Xizhao Chen, Lingfei Wan, Chunjie Liu, Yuting Yong, Yue Zhao, Shuli Sang, Lihua Zhang, Sheng Yao, Yushu Guo, Mingmei Wang, Xinhui Wang, Guangdun Peng, Xinlong Yan, Yanchun Wang, Min Zhang

**Affiliations:** ^1^ Guangzhou Institutes of Biomedicine and Health Chinese Academy of Sciences Guangzhou 510070 China; ^2^ Department of Pharmacy Medical Supplies Center Chinese PLA General Hospital Beijing 100853 China; ^3^ Beijing International Science and Technology Cooperation Base for Antiviral Drugs Beijing Key Laboratory of Environmental and Viral Oncology College of Chemistry and Life Science Beijing University of Technology Beijing 100124 China; ^4^ Department of Nephrology State Key Laboratory of Kidney Diseases National Clinical Research Center for Kidney Diseases First Medical Center Chinese PLA General Hospital Beijing 100853 China; ^5^ Laboratory of Advanced Biotechnology Beijing Institute of Biotechnology Beijing 100071 China; ^6^ Department of Pathology Fourth Medical Center Chinese PLA General Hospital Beijing 100048 China; ^7^ Department of General Surgery First Medical Center Chinese PLA General Hospital Beijing 100853 China

**Keywords:** cancer stem cells (CSCs), gastric cancer (GC), single cell RNA‐seq, spatial transcriptome, tumor microenvironment

## Abstract

Despite significant advancements in identifying novel therapeutic targets and compounds, cancer stem cells (CSCs) remain pivotal in driving therapeutic resistance and tumor progression in gastric cancer (GC). High‐resolution knowledge of the transcriptional programs underlying the role of CSC niche in driving tumor stemness and progression is still lacking. Herein, spatial and single‐cell RNA sequencing of 32 human gastric mucosa tissues at various stages of malignancy, illuminating the phenotypic plasticity of tumor epithelium and transcriptional trajectory from mature gastric chief cells to the CSC state, which is associated with activation of EGFR and WNT signaling pathways, is conducted. Moreover, the CSCs interact with not only the immunosuppressive CXCL13^+^ T cells and CCL18^+^ M2 macrophages to evade immune surveillance, but also the inflammatory cancer‐associated fibroblasts (iCAFs) to promote tumorigenesis and maintain stemness, which construct the CSC niche leading to inferior prognosis. Notably, it is uncovered that amphiregulin (AREG) derived from iCAFs promotes tumor stemness by upregulating the expression of SOX9 in tumor cells, and contributes to drug resistance via the AREG‐ERBB2 axis. This study provides valuable insight into the characteristics of CSC niche in driving tumor stemness and progression, offering novel perspective for designing effective strategies to overcome GC therapy resistance.

## Graphical abstract

A single‐cell and spatial transcriptional atlas reveals the architecture of human gastric cancer stem cell (CSC) niche and the developmental trajectory of CSCs. These CSCs interact with immunosuppressive immune cells thereby constructing immune‐suppressive niche, and inflammatory cancer‐associated fibroblast (iCAFs) to enhance stemness through upregulation of SOX9 and OLFM4, promoting drug resistance and proliferation via the AREG‐ERBB2 signaling pathway.

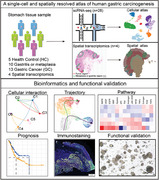

## 1 Introduction

Gastric cancer (GC) remains one of the leading causes of cancer‐related mortality worldwide. Despite significant advances in diagnostic and therapeutic strategies, the prognosis for advanced GC remains poor. Cancer stem cells (CSCs), a distinct subpopulation of undifferentiated tumorigenic cells, exhibit malignant phenotypic characteristics such as self‐renewal capacity and multi‐lineage differentiation potential, which are the primary drivers of tumor recurrence, drug resistance, and metastasis^[^ ^1^ ^]^ in GC. A comprehensive understanding of cellular and molecular characteristics of CSCs, as well as the interactions between CSCs and other components within tumor microenvironment (TME), is essential for elucidating the mechanisms that drive CSC‐mediated tumor recurrence, metastasis, and therapeutic resistance.^[^ ^2^, ^3^, ^4^, ^5^, ^6^, ^7^ ^]^

The origin of gastric CSCs remains controversial, which was thought to derive from the gastric mucosa stem and progenitor cells, or tumor cells that acquire stemness features undergoing epithelial‐mesenchymal transition (EMT),^[^ ^8^, ^9^ ^]^ suggesting the hierarchical and stochastic models of CSC formation, respectively. Previous studies have highlighted that the dysregulation of several signaling pathways, including the BMP/WNT, TGF‐β, PI3K/Akt, Notch, NF‐κB, and Hedgehog pathway, along with gene mutations, are crucial factors driving the uncontrolled proliferation of stem cells and their transformation into CSCs,^[^ ^8^, ^10^, ^11^, ^12^, ^13^, ^14^, ^15^ ^]^ supporting the hierarchical model. Conversely, other studies have demonstrated that reprogramming of differentiated cells can fuel repair, metaplasia, and neoplasia in the adult gastrointestinal tract,^[^ ^16^, ^17^ ^]^ suggesting the possibility of the stochastic model. Our recent research,^[^ ^18^ ^]^ along with others,^[^ ^19^, ^20^, ^21^, ^22^ ^]^ have reported that spasmolytic polypeptide‐expressing metaplasia (SPEM) arises from mature gastric chief cells via trans‐differentiation or dedifferentiation, which is critical in initiating preneoplastic and neoplastic lesions. Furthermore, lineage tracing studies have confirmed that differentiated cancer cells could revert to LGR5^+^ CSCs, thus contributing to tumor growth^[^ ^23^ ^]^ in colorectal cancer, while epigenetic regulation facilitates Lgr5^+^ intestinal stem cell formation by inducing the dedifferentiation of secretory precursors.^[^ ^24^ ^]^ Despite these advances, the phenotypic plasticity of tumor epithelium and transcriptional tumorigenic trajectory of gastric CSCs, as well as the mechanism by which the CSC niche induces gastric CSC formation, have not yet been fully elucidated.

CSCs reside within the specialized niche composed of immune cells, stromal cells and non‐cellular components such as extracellular matrix and soluble factors, which are essential for the maintenance, self‐renewal, differentiation, and plasticity of CSCs. The dynamic crosstalk between CSCs and their niche components plays an important role in modulating therapy resistance and recurrence. As the most abundant cellular components in the niche, cancer‐associated fibroblasts (CAFs) have been shown to drive tumor initiation, progression, therapy resistance, and metastasis. Su et al. reported that CD10^+^GPR77^+^ CAFs facilitate tumor formation and chemoresistance by creating a survival niche for CSCs.^[^ ^25^ ^]^ Ma et al. found that activation of cGAS‐STING signaling drive the SLC14A1^+^ CAF differentiation, which in turn confers stemness to bladder cancer cells via WNT5A paracrine pathway.^[^ ^26^ ^]^ Our group has demonstrated that CD146^+^ vascular CAFs could enhance tumorigenesis and stemness of intrahepatic cholangiocarcinoma cells via the IL‐6/IL‐6R axis.^[^ ^27^ ^]^ Although several studies have explored the mechanisms of GC initiation and progression, the intricate cellular and spatial architecture of the CSC niche driving GC progression requires further investigation.

The combined usage of single‐cell RNA sequencing (scRNA‐seq) and spatial transcriptomics has ushered in a revolutionary era in cancer research, providing an unprecedented avenue to dissect the cellular composition and molecular intricacies of TME with spatially resolved information across a wide range of human malignancies.^[^ ^28^ ^]^ Prior studies utilizing scRNA‐seq have successfully identified diverse cell populations within the GC TME, and revealed the transcriptomic regulatory network involved in the gastric metaplasia cascade, uncovering potential biomarkers for early identification of malignant cells.^[^ ^18^, ^29^ ^]^ Spatially resolved omics have further elucidated the spatial crosstalk between cancer cells and their surrounding immune and stromal cells, highlighting the critical role of immune infiltration and suppression in cancer progression.^[^ ^30^ ^]^ Nevertheless, the molecular characteristics and spatial distribution of gastric CSCs, along with the mechanisms by which the CSC niche induces cellular trans‐differentiation into gastric CSCs, remain incompletely understood.

In this study, we investigated the cellular and molecular landscape underlying the transition from normal gastric tissue to metaplasia and ultimately to cancer in the human stomach by integrating scRNA‐seq and spatial transcriptomic analysis. We achieved a comprehensive understanding of the molecular landscape of the components within TME, with a particular focus on identifying CSCs. Our results illuminated the phenotypic plasticity of tumor epithelium and transcriptional trajectory from mature gastric chief cells to the CSC state. We also discovered that CSCs interact with specific immunosuppressive immune cells thereby facilitating immune suppression. Importantly, we found that inflammatory CAFs (iCAFs) enhance tumor stemness by upregulating SOX9 and OLFM4, contributing to drug resistance and cell proliferation through the AREG‐ERBB2 signaling pathway. Our findings provide valuable insights into the heterogeneity of the CSC niche, emphasizing its role in driving tumor stemness and progression, and offer a foundation for developing targeted treatment strategies to overcome GC therapy resistance.

## 2 Results

### 2.1 A Dynamic Cellular Transcriptomic Landscape during Gastric Cancer Progression

**Figure 1:**
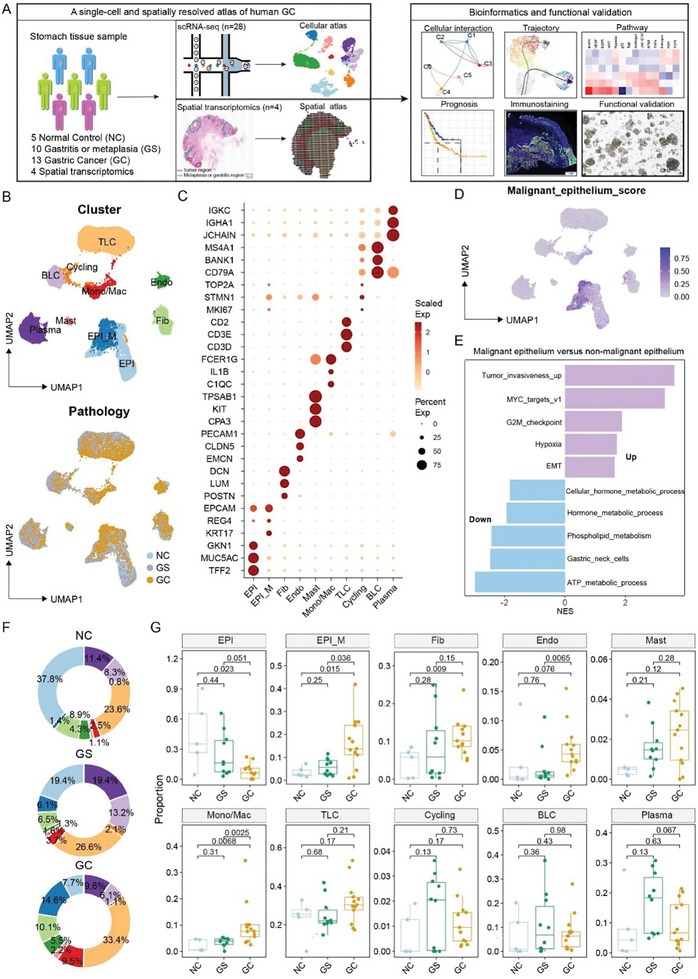
A single‐cell and spatially resolved transcriptomic atlas of human GC. A) The schematic diagram illustrates the research design in this study. A total of 28 GC biopsies were collected for single cell RNA sequencing, including three normal gastric mucosa tissues (NC), ten gastric mucosa tissues diagnosed with gastritis or metaplasia (GS), and 13 GC samples. Additionally, four GC samples underwent spatial transcriptomic sequencing and analysis, verified through multiplex immunofluorescence staining and functional experiments. B) UMAP plot displaying the distribution of 128, 940 high quality cells, colored by cell type (upper panel) and pathological lesions (lower panel). C) Dot plot showing the expression of representative marker genes for each cell type. D) UMAP plot illustrating the malignant epithelium scores across 128, 940 high quality cells. E) GO analysis demonstrating differential enrichment of biological processes between malignant and non‐malignant epithelium. F) Doughnut diagram representing the proportion distribution of cell types in NC, GS, and GC tissues. G) The boxplots depicting the relative proportions of each cell type across NC, GS, and GC tissues. P‐values were determined using Student's t‐test.

To decipher the dynamic molecular and cellular characteristics underlying GC initiation and progression, we first generated single‐cell transcriptome profiles from 28 human gastric mucosa samples at various stages of malignancy, originating from five normal control (NC) donors, ten patients with gastritis (GS), and 13 patients with GC (**Figure** **1**A; Table S1, Supporting Information). Additionally, spatial transcriptomics was performed to validate the scRNA‐seq findings in four GC samples across different tumor stages (Table S1, Supporting Information). All GC patients were treatment‐naive before undergoing tumor surgery. Viable cells, identified by 7‐aminoactinomycin D (7AAD) staining and sorted by fluorescence‐activated cell sorting (FACS), were isolated from each gastric mucosa sample for scRNA‐seq analysis. Following rigorous quality control, a total of 128  940 high‐quality cells were retained for further analysis, with median gene and unique molecular identifier (UMI) counts of 1 318 and 3 283, respectively (Figure S1A–C, Supporting Information). These cells were classified into ten major cell types based on the expression of specific cell‐type markers (Table S2, Supporting Information) and copy number variation (CNV) analysis (Figure 1B,C; Figure S1D,E, Supporting Information), including fibroblast (Fib, marked by DCN and LUM), endothelial cell (Endo, marked by PECAM1 and CLDN5), Mast cell (Mast, marked by TPSAB1 and CPA3), monocyte/macrophage (Mono/Mac, marked by IL1B and C1QB), T lymphoid cell (TLC, marked by CD3D/E and CD2), B lymphoid cell (BLC, marked by CD79A and MS4A1), Plasma cell (Plasma, marked by JCHAIN and IGHA1), Cycling cell (Cycling, marked by MKI67), non‐malignant epithelial cell (EPI, marked by TFF2 and GKN1), and malignant epithelial cell (EPI_M, marked by EPCAM and KRT17). We found that the malignant epithelial cells identified by inferCNV exhibited high malignant epithelium scores (Figure 1D), confirming their classification as EPI_M. Moreover, Gene Set Variation Analysis (GSVA) revealed that upregulated genes in EPI_M versus EPI were enriched in pathways related to tumor invasiveness, hypoxia, EMT, MYC target, and G2 M checkpoint, while downregulated genes were enriched in metabolic pathways (Figure 1E). The distribution of these ten major cell types across the 28 samples in the three sample groups is shown in Figure 1F. The percentage of each cell type varied significantly in these groups (Figure 1F; Figure S1F, Supporting Information), with an increase in the frequency of Endo, Mono/Mac, EPI_M, and Fib in GC compared to NC, while the proportion of non‐malignant EPI was decreased (Figure 1G). The odds ratios, calculated using the STARTRAC‐dist index (Ro/e) method, further validated the meta‐cluster cell distribution across the three sample groups (Figure S1G, Supporting Information).

### 2.2 Single Cell and Spatial Transcriptional Analysis of Cancer Stem Cell during GC Progression

**Figure 2:**
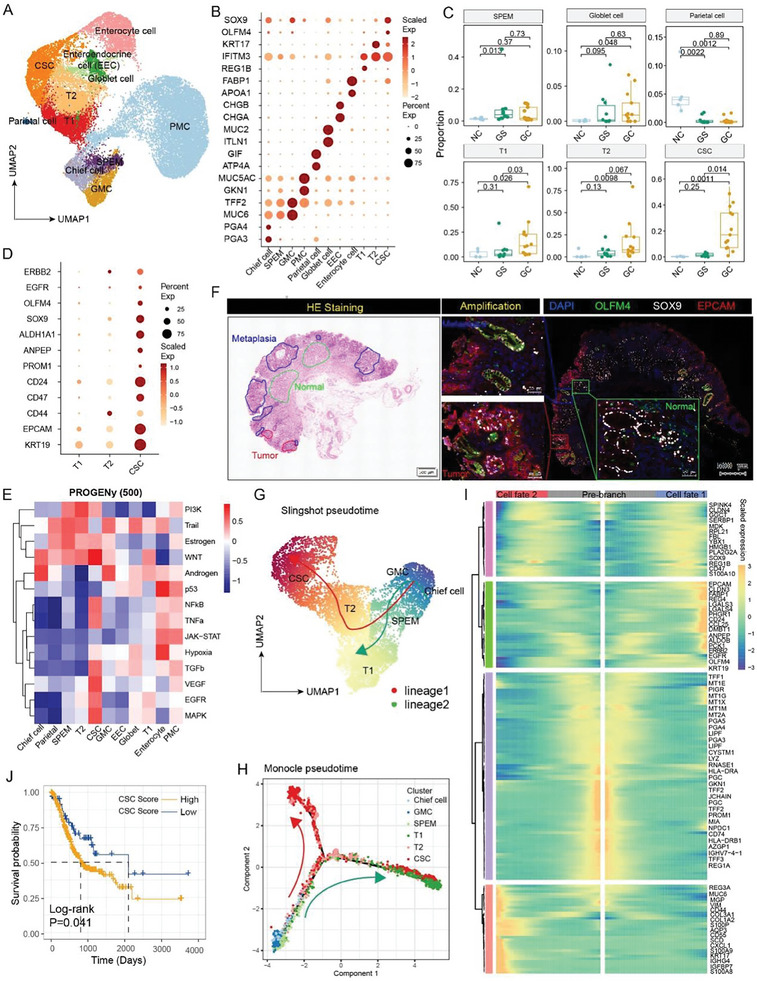
Characterization of CSCs during gastric carcinogenesis. A) Unbiased clustering of the entire epithelium identified eight non‐malignant epithelium subclusters: chief cells (Chief), pit mucous cells (PMC), gland mucous cells (GMC), parietal cells (Parietal), SPEM, goblet cells (Goblet), enterocyte cells (Enterocyte), and enteroendocrine cell (EEC), and three tumor cell populations: T1, T2, CSC. B) Dot plot displays the representative signature genes in each epithelium subclusters. C) The boxplots illustrate the relative proportions of each epithelium subcluster across three sample groups. P‐values were determined using Student's t‐test. D) Dot plot depicts the expression of specific stemness signature genes and cancer driver genes in T1, T2, and CSC. E) The heatmap displays the enrichment of signal pathways across different epithelium subsets, as inferred by Pathway Responsive Gene for Activity Inference (PROGENy) analysis using the top 500 genes. F) H&E and multiplex immunofluorescence staining featuring CSC markers including OLFM4 (green), SOX9 (white), and EpCAM (red) in various regions of GC slice. G,H) Pseudotime trajectory analysis showing the developmental trajectory of CSCs using Slingshot (G) and Monocle 2 (H). I) The heatmap illustrates the continuous changes of gene expression across different lineage trajectories. J) Kaplan‐Meier plot illustrating the correlation between CSC signature abundance and overall survival based on TCGA GC data, adjusted by age and tumor stage. P‐values were determined by the log‐rank test.

To characterize the dynamic spectrum of epithelium in the cascade from normal gastric mucosal tissue through gastritis to GC, we utilized Uniform Manifold Approximation and Projection (UMAP) analysis to decompose the epithelium into 11 main clusters. The epithelial clusters were annotated as follows: chief cell (marked by PGA3 and PGA4), pit mucous cell (PMC, marked by GKN1 and MUC5AC), gland mucous cell (GMC, marked by MUC6), parietal cell (marked by ATP4A and GIF), enterocyte cell (marked by APOA1), goblet cell (marked by MUC2), enteroendocrine cell (EEC, marked by CHGA), SPEM cell (marked by the median expression level of MUC6 and TFF2), and three subsets of malignant cells identified as T1 (marked by REG1B), T2 (marked by KRT17) and CSCs (marked by SOX9 and OLFM4), according to the expression of representative signature genes (**Figure** **2**A,B; Table S3, Supporting Information). The percentage of each epithelial cluster varied across three sample groups, the abundance of SPEM was prominently elevated in both GS and GC compared to NC, with higher abundance in GC, while the proportion of parietal cells gradually decreased in GS and GC. Additionally, the malignant epithelial clusters including T1, T2 and CSCs, showed higher percentage in GC (Figure 2C). These findings align with previous research^[^ ^18^ ^]^ indicating that preneoplastic gastric metaplasia is characterized by a transition from chief cells to SPEM, along with loss of parietal cells.

Further investigation was conducted to explore the molecular heterogeneity of malignant epithelium containing T1, T2, and CSCs. We found that CSCs expressed high levels of tumor stemness genes (PROM1, CD24, CD47, ANPEP, ALDH1A1, OLFM4, and SOX9), and demonstrated a high cancer stemness score (Figure S2A,B, Supporting Information), along with activation of the cancer driver genes EGFR and ERBB2 (Figure 2D). The Pathway RespOnsive GENes for activity inference (PROGENy) analysis confirmed that CSCs exhibited activation of inflammation pathways such as NFκB, TNFα, as well as stemness‐related signaling pathways such as WNT, hypoxia, EGFR, and TGF‐β signaling (Figure 2E; Figure S2C, Supporting Information). Moreover, multiplex immunofluorescence staining validated the existence of CSCs with co‐staining of SOX9, OLFM4, and EpCAM in the GC regions (Figure 2F). The Slingshot trajectory analysis revealed two major differentiation lineages. Lineage 1 indicated that SPEM cells originated from the trans‐differentiation of chief cells, and subsequently developed into T2 and CSCs (Figure 2G), which was validated by Monocle trajectory analysis (Figure 2H). The Monocle pseudo‐temporal dynamic expression pattern of signature genes also supported the trajectory where SPEMs developed into CSCs through trans‐differentiation of chief cells (Figure 2I). We also applied the CytoTRACE^[^ ^31^ ^]^ method to infer differentiation state and direction, discovering that CSCs exhibited the highest pluripotency score, while chief cells ranked the highest differentiation score. SPEM cells represented a transition status, displaying median levels of both pluripotency and differentiation scores (Figure S2D, Supporting Information). To further validate our findings, we reanalyzed the scRNA‐seq data of human gastric mucosa samples with different lesions and mouse gastritis samples induced by *Helicobacter pylori* infection or autoimmune gastritis.^[^ ^29^, ^32^ ^]^ These results validated that a subset of metaplasia cells expressed CSC‐related signature genes including SOX9, OLFM4, PROM1, and ANPEP (Figure S3 and S4, Supporting Information). The trajectory analysis among cell types from human pre‐malignant and early GC samples further support our hypothesis that SPEM originated from the trans‐differentiation of chief cells, and eventually developed into CSCs (Figure S4, Supporting Information). We further evaluated the association between the abundance of CSC and patient survival, and the Cox proportional hazards model was conducted, which included age and tumor stage. Our results showed that the frequency of CSC was negatively correlated with survival (P = 0.041, log‐rank test; Figure 2J), thereby predicting worse prognosis.

**Figure 3:**
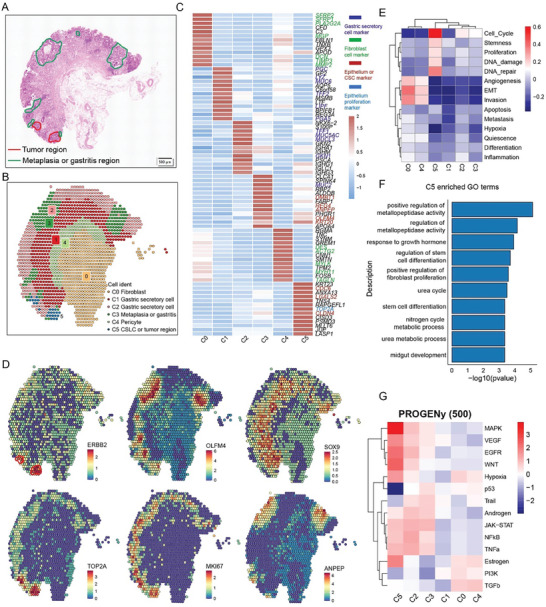
Validation of CSCs by spatial transcriptomics in early GC tissue. A) H&E staining of early GC tissue slices, including tumor, metaplasia or gastritis regions for spatial transcriptomic analysis. B) Unbiased clustering of spatial spots revealed six spatial subclusters and their identities. C) Heatmap showing the expression of the top 15 differential expressed genes (DEGs) across these six spatial subclusters. D) The spatial expression patterns of CSC maker genes are displayed in spots, with color gradients from blue (low expression) to red (high expression). E) The heatmap illustrating the enrichment of signal pathways in different cell types based on spatial transcriptomics. F) GO term analysis revealing enriched biological process in C5 or CSC regions. G) Heatmap displaying the enrichment of signal pathways in different subsets, as determined by PROGENy analysis using the top 500 genes.

To characterize and visualize the spatial distribution of CSCs in situ, we further performed spatial transcriptomic profiling on early and advanced GC tissues (**Figure** **3**A; Figure S5, Supporting Information). First, dimensionality reduction clustering of the early GC spatial transcriptomic data revealed six spatial clusters (C0‐C5, Figure 3B). The spatial clusters C1 and C2 predominantly expressed marker genes associated with the secretion of gastric mucus and digestive enzymes, such as MUC5AC, GKN1, PGC, and LIPF. Spatial clusters C0 and C4 mainly expressed fibroblast and pericyte associated markers such as SFRP1, DCN, TAGLN, and MYH11 (Figure 3C). Notably, the spatial distribution of spatial clusters C0/1/2/4 was strictly localized in the normal gastric mucosa regions (Figure 3B). Interestingly, the spatial clusters C3 and C5 both expressed high levels of stem cell markers OLFM4, SOX9, and ANPEP, corresponding to the metaplasia and tumor regions, respectively. Additionally, we observed that the cell proliferation markers MKI67 and TOP2A were highly expressed in spatial cluster C5, which was exclusively located in the tumor region (Figure 3D). Cancer signaling pathway analysis (Figure 3E), Gene Ontology (GO) term analysis (Figure 3F), and PROGENy analysis (Figure 3G) revealed that spatial cluster C5 was enriched with CSC‐related signal pathways. These results were also validated in advanced GC, where the spatial cluster C8 expressed CSC‐related markers OLFM4, SOX9, PROM1, and ANPEP, as well as tumor proliferation marker MKI67 and tumor driver gene ERBB2 (Figure S5, Supporting Information).

### 2.3 Suppressive Immune Cells and Their Interaction with CSCs during GC Progression

**Figure 4:**
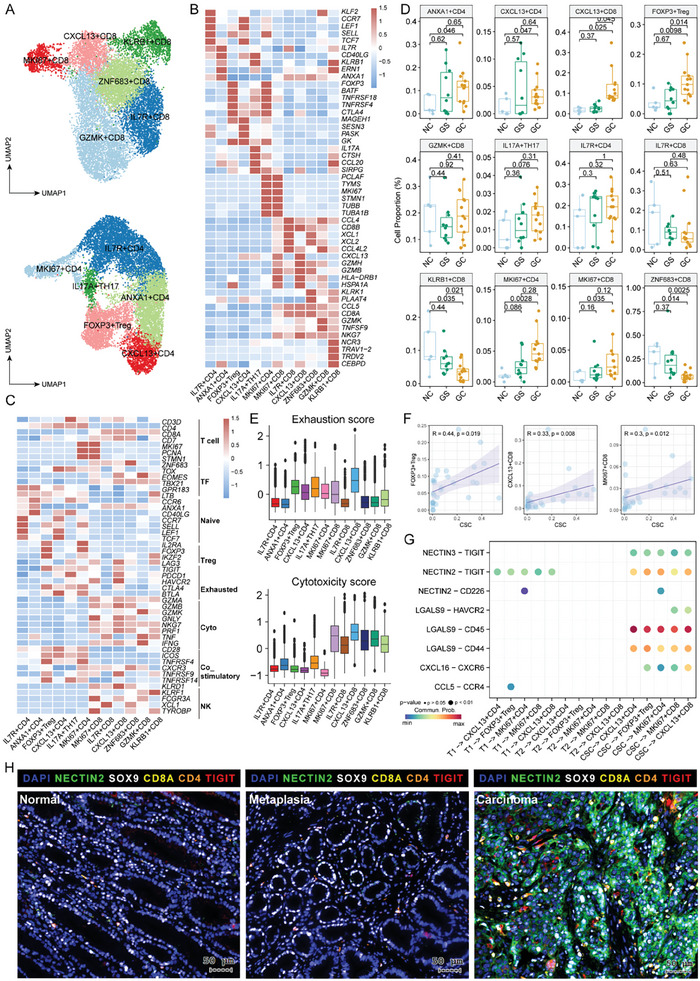
Molecular and cellular characterization of T subsets during GC carcinogenesis. A) The UMAP showing subsets of CD8 T cells (upper panel) and CD4 T cells (lower panel). B) The heatmap depicts the expression of top five DEGs among different T subsets. C) The heatmap depicts the expression patterns of functional signatures within each T cell subset. D) The boxplot illustrates the changes in the relative proportions of each T cell subset across three sample groups, p‐values were calculated using two‐sided Student's t‐test. E) The boxplot displays the enrichment of signatures related to exhaustion (upper) and cytotoxicity (bottom) in different T cell subsets. F) The dot plot illustrates the correlation coefficients between CSCs and CXCL13^+^ CD8 T cells, MKI67^+^ CD8 T cells, and FOXP3^+^ Treg cells. The oblique line and R represent the average correlation coefficient. G) Bubble plot displays the ligand‐receptor pairs involved in the interactions between different T cell subsets and tumor cells (T1, T2, and CSCs). H) Multiplex immunofluorescence staining of T cells (CD4, orange; CD8, yellow), CSCs (SOX9, white; NECTIN2, green), immune checkpoint molecular (TIGIT, red), and nuclear stain DAPI (bule), in NC (left), metaplasia (middle), and GC (right). The staining elucidates the interactions between T cells and epithelium mediated through TIGIT‐ NECITIN2.

Alongside epithelial cells, the tumor‐infiltrating lymphocytes (TILs) and myeloid cells play crucial roles in shaping the TME. To investigate the molecular characteristics of key immune subclusters involved in GC progression, we initially divided TILs into 12 subclusters (**Figure** **4**A), with distinct gene expression patterns (Figure 4B,C; Tables S4 and S5, Supporting Information). These subclusters included naïve IL7R^+^ CD8 T cell, resident ZNF683^+^ CD8 T cell, proliferative MKI67^+^ CD8 T cell, GZMK^+^ CD8 T cell, CXCL13^+^ CD8 T cell, KLRB1^+^ CD8 T cell, naïve IL7R^+^CD4 T cell, proliferative MKI67^+^ CD4 T cell, IL17A^+^ Th17, FOXP3^+^ Treg, ANXA1^+^ CD4 T cell, and CXCL13^+^ CD4 T cell. Comparison of the relative proportions of each TILs subcluster across different patient groups revealed that the CXCL13^+^ CD8, FOXP3^+^ Treg, and MKI67^+^ CD8 T cells were remarkably enriched in GC compared to GS and NC, while the proportion of ZNF683^+^ CD8 decreased during GC progression (Figure 4D). CXCL13^+^ CD8 T cells, FOXP3^+^ Tregs, and MKI67^+^ CD8 T cells exhibited high expression levels of T cell exhaustion marker genes. Notably, FOXP3^+^ Tregs and MKI67^+^ CD8 T cells displayed high exhaustion signature scores, while CXCL13^+^ CD8 T cells exhibited both high exhaustion and cytotoxicity signature scores (Figure 4D,E), indicating an immunosuppressive identity. Interestingly, we found notable positive correlations between the abundance of the CXCL13^+^ CD8 T cells, FOXP3^+^ Tregs, and MKI67^+^ CD8 T cells with CSCs (Figure 4F). Importantly, intercellular crosstalk analysis revealed that CSCs frequently interacted with the immunosuppressive TILs, including CXCL13^+^ CD8, FOXP3^+^ Tregs, and MKI67^+^ CD8 T cells, through the NECITIN2/3‐TIGIT and LGALS9 (Galectin‐9)‐HAVCR2 ligand‐receptor pairs (Figure 4G), which constitute the immune checkpoint signaling pathways leading to immune suppression.^[^ ^33^, ^34^, ^35^ ^]^ Additionally, multiplex immunofluorescence staining of gastric mucosa samples from NC, GS, and GC tissues validated that SOX9‐positive CSCs could interact with T cells through NECITIN2‐TIGIT axis, providing potential insights into the mechanism by which CSCs evade T cell‐mediated immune surveillance (Figure 4H).

We also identified eight myeloid subclusters including VEGFA^+^ monocyte (VEGFA^+^ Mono), CPA3^+^ mast cell (CPA3^+^Mast), two types of dendritic cells (DC): CD1C^+^DC and LAMP3^+^DC, three types of tumor‐associated macrophages (TAM): CCL18^+^TAM, FOLR2^+^TAM, and SLC40A1^+^TAM, and CXCR2^+^ neutrophil cell (CXCR2^+^Neu), characterized by distinct gene expression and transcription factor patterns (Figure S6A–C and Table S6, Supporting Information). Comparing the proportions of myeloid subclusters among different groups revealed that LAMP3^+^ DCs and CCL18^+^ TAMs were enriched in GC groups (Figure S6D,E, Supporting Information). Additionally, CCL18^+^ TAMs highly expressed M2‐type macrophage features including FN1, CTSB, CSTL, CTSD, MMP9, and MRC1 (Figure S6F, Supporting Information), as well as metabolite‐associated features including FABP5 and APOE (Figure S6B, Supporting Information), indicating their anti‐inflammatory and pro‐tumorigenic properties. Notably, we observed significant positive correlations between the abundance of CCL18^+^ TAMs, CXCL13^+^ CD8 T cells, and CSCs (Figure S6G, Supporting Information). Further intercellular crosstalk analysis revealed that CCL18^+^ TAMs mainly interacted with CSCs through THBS1‐CD47, TNFSF12‐TNFRSF12A, and FN1‐CD44 signaling axes (Figure S6H,I, Supporting Information). CCL18^+^ TAMs also interacted with MKI67^+^ CD8, CXCL13^+^ CD8, and CXCL13^+^ CD4 T cells through NECITIN2‐TIGIT and LGALS9‐HAVCR2 pathways (Figure S6H, Supporting Information), which are associated with T cell suppressive features and immune escape. Furthermore, survival analysis revealed that the enrichment of CCL18^+^TAM was associated with an unfavorable prognosis (Figure S6J, Supporting Information).

### 2.4 The Dynamic Characteristics of Cancer‐Associated Fibroblasts during GC Progression

**Figure 5:**
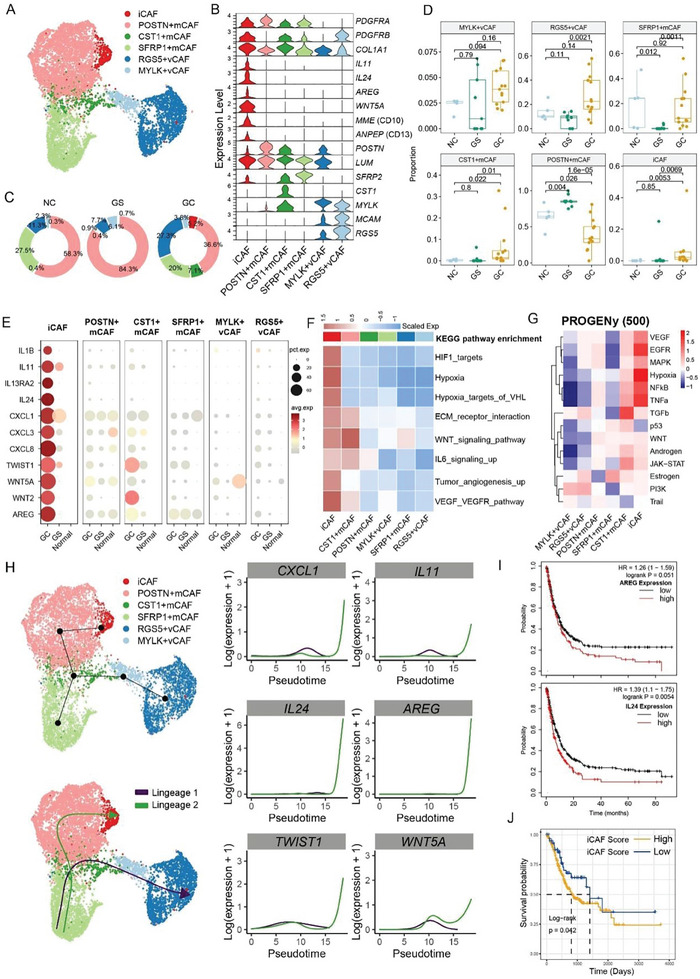
Molecular and cellular characterization of CAF subsets during GC carcinogenesis. A) Sub clustering of CAFs reveals six distinct subsets, illustrated by the UMAP plot. B) The stacked violin plot depicts the expression patterns of signature genes in distinct CAF subsets. C,D) Distribution of CAF subsets across different gastric lesions is represented by the pie chart (C) and quantified in box plots (D). E) Bubble plot illustrating the expression of cytokines, chemokines, and genes related to WNT signaling pathways in CAF subsets across different gastric lesions. F,G) The heatmap illustrates the enrichment of signaling pathways in different CAF subsets, analyzed by GSVA (F) and PROGENy using the top 500 genes (G). H) Pseudotime trajectory analysis reveals the lineage relationships among different CAF subsets (left panel), and highlights the dynamic expression patterns of signature genes enriched in the iCAFs (right panel). I) The Kaplan‐Meier survival curves illustrate the correlation between overall survival and the relative expression of AREG (upper panel), IL24 (middle panel) and iCAF signatures (bottom panel) in TCGA GC data, adjusted by age and tumor stage. P values were calculated using the log‐rank test.

Sub‐clustering of CAFs revealed six subpopulations: inflammatory CAF (iCAF, marked by IL11, IL24, MME (CD10), ANPEP (CD13)), POSTN^+^ matrix CAF (POSTN+ mCAF, marked by LUM and POSTN), CST1^+^ matrix CAF (CST1+ mCAF, marked by LUM and CST1), SFRP1^+^ matrix CAF (SFRP1+ mCAF, marked by LUM and SFRP1), MYLK^+^ vascular CAF (vCAF, marked by MACM and MYLK), and RGS5^+^ vascular CAF (RGS5+vCAF, marked by MACM and RGS5), according to the expression patterns of specific genes, DEGs, and transcriptional factors (TFs). (**Figure** **5**A,B; Figure S7A,B and Table S7, Supporting Information). Analysis of the relative proportions of each CAF subcluster indicated that iCAFs and CST1^+^mCAFs were highly enriched in GC compared to GS and NC (Figure 5C,D). iCAFs expressed high expression levels of inflammatory cytokines and chemokines (IL1B, IL11, IL13RA1, IL24, and CXCL1/3/8), as well as growth factors including WNT2/5 and amphiregulin (AREG), with even higher expression observed in iCAFs derived from GC compared to NC and GS (Figure 5E). The KEGG (Figure 5F) and PROGENy (Figure 5G) analysis of different CAF subtypes unveiled that iCAFs were enriched in the IL6 signal pathway, hypoxia, and tumor growth signals, including EGFR, WNT, MAPK, and TGF‐β, all of which are critical for CSC maintenance.^[^ ^36^ ^]^ Additionally, our results highlighted the involvement of tumor angiogenesis related signaling pathway VEGF‐VEGFR in both iCAFs and CST1^+^ mCAFs (Figure 5F), indicating their important roles in promoting tumor invasion and metastasis. The trajectory analysis among the CAF clusters demonstrated that iCAFs originated from CST1^+^mCAFs through POSTN^+^mCAFs, while the other branch demonstrated the transition of RGS5^+^vCAFs from CST1^+^mCAFs through MYLK^+^vCAFs (Figure 5H). The transcriptomic feature similarity analysis (Figure S7C, Supporting Information) and differentiation trajectory analysis (Figure 5H; Figure S7D, Supporting Information) demonstrated that CST1^+^mCAFs represented the transitional status which determined the cellular differentiation routes. The pseudo‐temporal dynamic expression pattern analysis demonstrated upregulation of iCAF signature genes, such as IL11, IL24, CXCL1, AREG, TWIST1, and WNT5A, aligning with differentiation trajectory analysis (Figure 5H; Figure S7E, Supporting Information). Survival analysis further revealed that the expression of iCAF signature genes AREG and IL24, as well as the abundance of iCAFs, predicted a worse prognostic outcome (Figure 5I,J).

### 2.5 Interaction of iCAFs with CSCs Involved in GC Progression

**Figure 6:**
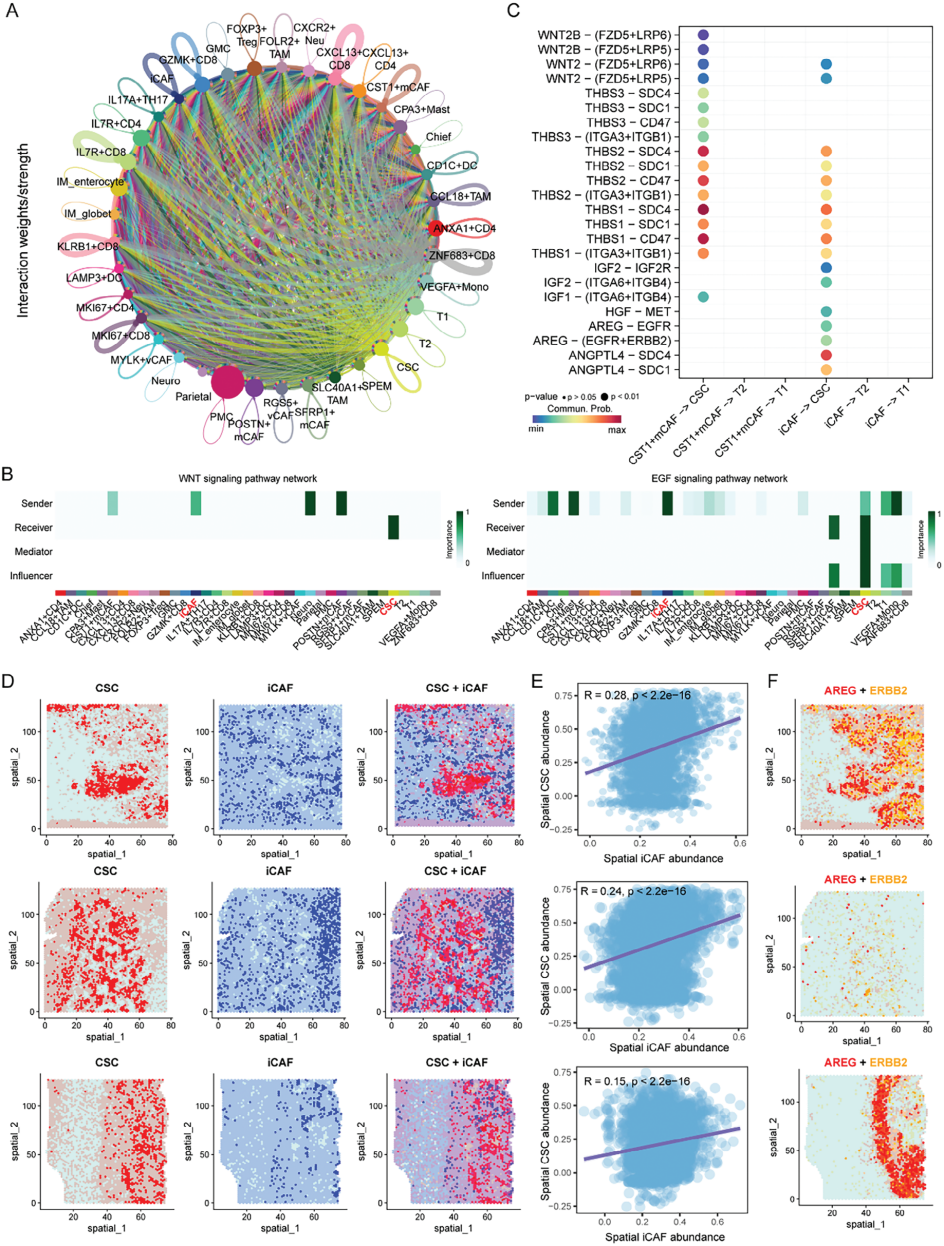
Single‐cell and spatial profiling revealed cellular interactions within CSC niche. A) The circle plot depicts global interaction strength among cellular components within the CSC niche by scRNA‐seq. Color intensity indicates the strength of the interactions. B) The heatmap illustrates the involvement of WNT (left) and EGF (right) signaling pathways in the interactions between CSCs and their niche by scRNA‐seq. C) The ligand‐receptor pairs mediating interactions between CSCs and their principal CAF niche by scRNA‐seq. D) Spatial distribution of CSCs and iCAFs in three GC samples by spatial transcriptomic sequencing (10x Genomics Visium). E) The correlation of spatial distribution of iCAFs and CSCs in three GC samples by 10x Genomics Visium. F) The co‐expression of AREG and ERBB2 in three GC samples by 10x Genomics Visium.

A comprehensive and systemic investigation of the cellular communication among different cell types within GC TME was conducted and demonstrated that iCAFs and CST1^+^ mCAFs frequently interacted with immune cells and tumor cells, especially the CSCs (**Figure** **6**A). Consistent with these above results, we observed that iCAFs interacted with CSCs through the WNT and EGF signaling pathways (Figure 6B; Figure S7F,G, Supporting Information). Intercellular crosstalk analysis highlighted that ligand‐receptor pairs, including WNT2‐FZD5/LRP6, WNT2‐FZD5/LRP5, THBS2‐SDC1/SDC4/CD47, IGF2‐(ITGA6+ITGB4), IGF1‐(ITGA6+ITGB4), ANGPTL4‐SDC1/SDC4, HGF‐MET, and AREG‐EGFR/ERBB2, were enriched in the intercellular crosstalk between iCAFs and CSCs (Figure 6C). Spatial transcriptomic results further confirmed the co‐existence and positive correlation between iCAFs and CSCs in both early and advanced stage GC (Figure 6D,E). Additionally, AREG expression spatially aligned with ERBB2 in situ (Figure 6F), which is coincided with the spatial locations of both iCAFs and CSCs, indicating that the interactions between iCAFs with CSCs were guided by the AREG‐ERBB2 axis.

### 2.6 iCAFs Remarkably Promoted GC Proliferation and Stemness through AREG‐ERBB2 Axis

**Figure 7:**
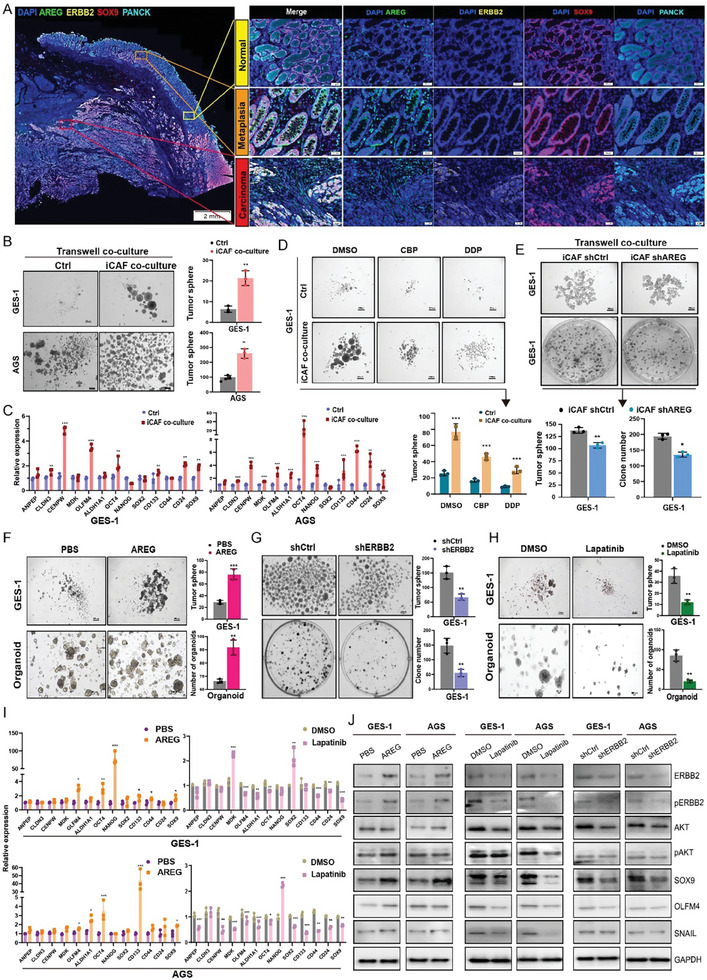
iCAFs promoted GC proliferation and stemness through the AREG‐ERBB2 pathway. A) Multiplex immunofluorescence staining of PanCK, SOX9, ERBB2, AREG, and DAPI was performed in the distinct gastric regions exhibiting characteristics of normal, metaplasia, and cancer. Scale bars: 2 mm or 50 µm. B) Representative images demonstrating that iCAFs significantly enhanced sphere formation via a transwell co‐culture system in vitro. Scale bars: 200 µm. C) The expression levels of CSC‐associated marker genes were analyzed by qRT‐PCR following co‐culture of GC cells with iCAFs. D) iCAFs markedly induced chemotherapy resistance in GC cells during sphere formation in transwell co‐culture. E) Lentivirus‐mediated AREG knockdown (shAREG) in the iCAFs substantially reduced their capacity to promote tumor sphere and clone formation in GC cells. F) Tumor sphere formation of GC cells and organoids from GC patients were treated with AREG (50 ng mL^−1^) or PBS control, respectively. G) Lentivirus‐mediated ERBB2 knockdown (shERBB2) in GES‐1 cells were further evaluated through tumor sphere and clone formation assays. Scale bars: 200 µm. H) Tumor sphere formation of GES‐1 cells and organoids were treated with Lapatinib (10 µM, a specific small molecular inhibitor of ERBB2) or DMSO as a control. Scale bars: 200 µm or 100 µm. I) The expression of CSC‐related marker genes was assessed by qRT‐PCR analysis, when GC cells were treated with AREG (50 ng mL^−1^), or Lapatinib (10 µM) for 48 h, respectively. J) Western blotting analysis of ERBB2, pERBB2, SOX9, OLFM4, SNAIL, AKT, and p‐AKT in GC cells treated with AREG (50 ng mL^−1^), Lapatinib (10 µM), or shERBB2, respectively. *P < 0.05, **P < 0.01, and ***P < 0.001 (two‐sided unpaired t test).

To further verify the enrichment of AREG expression in the iCAF subcluster of GC tissues, we conducted multiplex immunofluorescence staining on whole gastroscopy sections, including normal, metaplasia, and GC tissues. Our results showed that GC tissues exhibited a remarkable enrichment of the AREG^+^ iCAFs and SOX9^+^ CSCs, compared to normal and metaplasia tissues (**Figure** **7**A). Subsequently, we isolated iCAFs from GC tissues using FACS‐based enrichment of PDGFRa and CD10, and expanded as previously reported.^[^ ^37^ ^]^ Transwell co‐culture assays indicated that iCAFs significantly promoted tumor sphere formation in GC cells (Figure 7B). Moreover, qRT‐PCR analysis showed a substantial increase in the expression levels of CSC‐related markers in GC cells when co‐cultured with iCAFs (Figure 7C). Notably, the presence of iCAFs also induced drug resistance in GC cells, as evidenced by tumor sphere formation (Figure 7D; Figure S8A, Supporting Information) and colony formation assays (Figure S8B, Supporting Information) following chemotherapeutic drug treatment. Additionally, AREG depletion in iCAFs significantly suppressed their tumor‐promoting effects on tumor sphere and clone formation (Figure 7E; Figure S8C,D, Supporting Information), while exogenous AREG administration markedly promoted tumor sphere and organoid formation (Figure 7F). Conversely, the depletion of AREG receptor ERBB2 in GC cells significantly reduced their tumor sphere formation capacity (Figure 7G). These findings were further supported using the ERBB2‐specific inhibitor, Lapatinib, which yielded comparable results in GC cells and organoids (Figure 7H). To investigate the downstream mechanism of the AREG‐ERBB2 axis in GC cells, we conducted qRT‐PCR analysis and observed that AREG treatment significantly increased the expression of CSC markers such as OCT4, CD133, OLFM4, and SOX9. Conversely, treatment with Lapatinib significantly repressed the expression of the CSC‐related genes such as OCT4, OLFM4, and SOX9 (Figure 7I). Furthermore, Western blotting assays revealed that AREG enhanced the expression of ERBB2 and p‐ERBB2, and downstream proteins including SOX9, OLFM4, SNAIL, AKT, and p‐AKT. In contrast, the depletion of ERBB2 or treatment with lapatinib in GC cells markedly suppressed the expression of these corresponding proteins (Figure 7J). Taken together, these findings suggest that the AREG‐ERBB2 axis functions as a crucial ligand‐receptor pair in promoting tumorigenesis, by directly connecting iCAFs to tumor cells through AKT‐mediated cancer stemness and proliferation.

## 3 Discussion

Despite significant advances in targeted cancer therapies, drug resistance and tumor progression driven by CSCs remain major challenges in clinical management of GC. A comprehensive analysis of the cellular and molecular changes during the progression from normal tissue to metaplasia and eventually to cancer in the human stomach is critical for understanding how the CSC niche drives tumor progression. Herein, by integrating unbiased scRNA‐seq and spatial transcriptomics, we elucidated the phenotypic plasticity of tumor epithelium and the transcriptional tumorigenic trajectory from SPEM to CSCs via trans‐differentiation of chief cells. By focusing on gastric CSCs, we gained critical insights into their origin, molecular characteristics, spatial distribution, and interactions within the CSC niche. We discovered significant correlations and interactions between CSCs and specific immunosuppressive immune cell subsets, including CXCL13^+^ T cells, CCL18^+^ TAMs, which are essential to evade immune surveillance. Notably, we also found that IL11^+^CD10^+^ iCAFs were a key component of CSC niche that drive tumor stemness in CSCs by upregulating the expression of SOX9 and OLFM4, leading to drug resistance via the AREG‐ERBB2 signaling pathway.

Tumor initiation has been reported to be driven by either tissue stem cells or transformed differentiated cells, suggesting the prevailing hierarchical and stochastic models. In the hierarchical model, tumor cells originate from the tissue stem cells with abnormal growth control, eventually transforming into CSCs that lead to tumorigenesis and metastasis.^[^ ^38^, ^39^ ^]^ Recent studies have validated the presence of tissue resident stem cells in gastric tissues by integrating lineage tracing and scRNA‐seq, revealing that LEFTY1 can be identified as novel stem cell marker within gastric mucosa and isthmus stem cells exhibiting active cycling features can maintain long‐term self‐renewal capacity, further governing the turnover of pit‐isthmus‐neck within the corpus gland.^[^ ^40^, ^41^ ^]^ Conversely, the stochastic model implied that tumor cells can trans‐differentiate into CSCs through genetic or epigenetic changes. Recently, the metaplasia cell lineage, represented the transition cell types, had also been demonstrated to be the origin of preneoplastic cells.^[^ ^19^, ^20^, ^21^, ^22^ ^]^ Stella et al., reported that a cancer‐like metaplastic subtype expressing ANPEP shared transcriptional features with GC, indicating the carcinogenic capacity.^[^ ^26^ ^]^ Our scRNA‐seq data alongside with the scRNA‐seq data of Zhang^[^ ^23^ ^]^ et al., also demonstrated that the MUC6^+^TFF2^+^ SPEM cells arise from the trans‐differentiation of mature chief cells, and two trajectory prediction methods revealed that SPEM cells could further develop into CSCs. Additionally, the multiplex immunofluorescence staining and spatial transcriptomics confirmed the existence of CSCs in metaplasia and tumor regions.^[^ ^42^ ^]^ The enrichment of cell cycle and VEGF signaling pathways in CSCs demonstrates their high capacity of proliferation and angiogenesis, suggesting that the fundamental mechanism of CSCs in cancer progression is attributed to their roles in maintaining the clonal population of tumor cells and facilitating tumor angiogenesis. The existence of these two models may be due to the varying pathological lesions and anatomical locations of gastric tissues in different studies, highlighting the heterogeneity of gastric tumorigenesis.^[^ ^43^ ^]^ More precise and specific markers of CSCs are urgently needed for in vivo lineage tracing to track their origin and contribution during gastric carcinogenesis.

The interplay between CSCs and their niche has been implicated in tumor metastasis, recurrence, heterogeneity, and therapeutic resistance. We next focused on the role of CSC niche in regulating CSCs during GC progression. We found that CSCs, highly expressed immune checkpoint molecules such as NECTIN2/3 and LGALS9, interacted with suppressive CXCL13^+^ T cells through the ligand‐receptor pairs: TIGIT‐NECTIN2/3 and LGALS9‐HAVCR2, which contribute to the increase of Tregs and inhibition of the activation and proliferation of cytotoxic T lymphocytes, thus providing an immunosuppressive niche that is critical to tumor cell immune escape. Besides, we found that the abundance of CSCs was positively correlated with anti‐inflammatory CCL18^+^ TAMs, which expressed TNFSF12, THBS1, and FN1. The presence of CCL18^+^TAMs and VEGFA^+^TAMs is significantly higher in GC tissues compare to NS and GS. These TAMs not only facilitate immune escape by interacting with CSCs through the TIGIT‐NECITIN2 and LGALS9‐CD44/45, but also contribute to metastasis by secreting angiogenesis‐related signal molecules including TNF and TGFβ. Alongside the immune cells, CAFs, the major component of TME, not only secrete cytokines and growth factors to enhance CSC survival and self‐renewal, but also contribute to extracellular matrix remodeling and angiogenesis, facilitating tumor metastasis and therapy resistance.^[^ ^44^ ^]^ Prior studies have revealed that CAFs secrete CXCL16 and TGFβ to promote the acquisition of EMT‐driven tumor stemness.^[^ ^45^ ^]^ The CD10^+^GPR77^+^ CAFs induce CSC self‐renewal and chemoresistance by secreting IL‐6 and IL‐8.^[^ ^46^ ^]^ CAFs also serve as a CSC niche, promoting cancer stemness in bladder cancer, and facilitating symmetric division and dedifferentiation of ovarian cancer cells through noncanonical Wnt5a signaling. Meanwhile, CAFs enhance pancreatic ductal adenocarcinoma clonogenic (PDAC) growth, and promote CSC self‐renewal and proliferation by producing type I collagen that activates the integrin‐FAK signaling pathway.^[^ ^47^ ^]^ Herein, we demonstrated that iCAFs highly expressed inflammatory chemokines and cytokines, including IL1B, IL11, IL24, CXCL1, CXCL3, and CXCL8, and are enriched in WNT, VEGF, EGFR, and tumor angiogenesis signaling pathways, which establish a niche that supports the maintenance and invasion of CSCs. Integrating scRNA‐seq and spatial transcriptomic analysis, we demonstrated that iCAFs highly interacted with CSCs through AREG‐ERBB2 axis, which has previously been reported to be induced by TGFβ in myofibroblasts to promote PDAC metastasis.^[^ ^48^ ^]^ Considering that targeting AREG derived from senescent stroma can attenuate cancer resistance and reverse PD‐L1 mediated immunosuppression,^[^ ^49^ ^]^ a combinational treatment targeting AREG and CSCs may potentially provide an effective approach for treating GC.

While these data represent an important advance in knowledge, future studies should consider the limitations of the current work. First, the resolution of spatial transcriptomic sequencing restricted us to visualize the distribution of CSCs and their interactions with neighboring cells at single cell level. Second, while we have identified two CSC markers including SOX9 and OLFM4 at single‐cell resolution and in vitro experiments, in vivo functional validation in animal models to confirm the role of CSCs in tumor initiation, progression, and resistance is still required. However, SOX9 and OLFM4 also were lowly expressed in normal epithelium cells, the routine used single Sox9‐cre or Olfm4‐cre mediated Tdtomato or mCherry labeling for lineage tracing in mouse GC models cannot distinguish their fates of differentiation or trans‐differentiation. Due to the longtime induction of mouse GC models and the complexity of replicating human GC in vivo, and the limitation of the unspecific Sox9‐cre or Olfm4‐cre genetic tracing in vivo, alternative genetic strategies are needed to specifically label the trans‐differentiation of gastric CSCs in vivo, such as the dual recombinase‐mediated intersectional genetic strategies using combined Dre‐rox and Cre‐loxP system. Third, our proposed model suggests that CSCs originate from the trans‐differentiation of chief cells; however, the limited sample size prevents us from ruling out the contribution of tissue‐resident stem cells from the isthmus at early stages. The complexity of epithelial cell metaplasia and trans‐differentiation process pose challenges in identifying specific markers for in vivo lineage tracing to determine the origin of gastric CSCs. Inducible lineage tracing experiments using specific markers, combined with continuous endogenous markers, such as mitochondrial DNA (mtDNA) mutation, DNA methylation epimutation, and CNV, are essential to track the formation of CSCs in vivo.

In conclusion, we have constructed a comprehensive single‐cell and spatial transcriptomic atlas of CSCs and their niche in GC, detailing the origins, characteristics of CSCs, and the interactions within CSC niche. Our study significantly deepened the understanding of the cellular and molecular mechanism of CSC niche in regulating the CSC self‐renewal, drug resistance and tumor progression. These findings pave the way for the development of novel diagnostic tools and targeted therapies, ultimately aiming to improve outcomes for patients suffering from GC.

## 4 Experimental Section

### Ethics Approval

The research was executed in adherence to the ethical principles outlined in the Declaration of Helsinki and received clearance from the Ethics Review Board of the Fourth Medical Center at PLA General Hospital, under the approval numbers 2021KY011‐HS001 and 2021KY041‐HS001. Prior to participation, all individuals involved in the study provided their written consent, indicating their informed agreement to participate.

### Acquisition of Fresh Tissue Materials and Preparation of Single Cell Suspension

The freshly excised samples were rinsed with phosphate‐buffered saline (PBS) and separated into two sections. One section was processed for single‐cell suspension, while the other section was reserved for additional experiments, encompassing 10X Visium spatial transcriptomics, hematoxylin and eosin (H&E) staining, and immunofluorescence staining. Immediately after collection, the surgical samples intended for single‐cell suspension preparation were immersed in a tissue preservation solution (Miltenyi Biotec, Germany) and transported on ice bath for rapid processing. The fresh tissue samples were rinsed three to four times with prechilled Dulbecco's Phosphate‐Buffered Salines (DPBS, Solarbio, Beijing) at 4 °C. Using surgical scissors, the tissues were dissected into small fragments and transferred to a 1.5 mL centrifuge tube. These tissue fragments were then incubated at 37 °C for 30–50 min in either a custom enzymatic digestion solution (1 mg mL^−1^ type IV collagenase (Solarbio, Beijing) and 10 U µL^−1^ DNase I (Roche)) or a commercial MACS Human Tumor Dissociation kit (DS_130‐095‐929, Miltenyi Biotec, Germany). Digestion was stopped when the tissue appeared fully dissociated and turbid. The resulting cell suspension was filtered through a 40‐µm cell strainer and centrifuged at 300 rpm for 5 min at 4 °C. After discarding the supernatant, the cells were washed in 1 mL of DPBS and treated with 3 mL of precooled red blood cell lysate (Solarbio, Beijing). The mixture was incubated for 5–10 min at 4 °C before another centrifugation. Subsequently, the cells were stained with 7‐Aminoactinomycin D (7‐AAD, eBiocience, Cat# 00‐6993‐50) staining solution at 25 °C for 5 min. High‐quality individual cells were then sorted using a BD Aira II flow cytometer (FACS), and cell viability was confirmed under a microscope using trypan blue staining prior to constructing the single‐cell transcriptomic library.

### Single‐Cell RNA Sequencing

The scRNA‐seq and TCR libraries were generated using a 10X Genomics Chromium Controller platform, along with Chromium Single Cell 5′ library & gel bead kit in Capitalbio Technology Corporation (China). In brief, cells were concentrated to ≈1000 cells/µL and loaded into each channel to produce single‐cell gel bead‐in‐emulsions (GEMs). After the reverse transcription step, the GEMs were disrupted, and barcoded complementary DNA (cDNA) was collected and amplified. The amplified barcoded cDNA was subjected to fragmentation, adaptors ligation and index PCR amplification. The concentration of final libraries was quantified by the Qubit High Sensitivity DNA Assay (Thermo Fisher Scientific). The size distribution of the libraries was analyzed using a High Sensitivity DNA chip with a Bioanalyzer 2200 (Agilent). All the libraries were sequenced using NovaSeq 6000 (Illumina, San Diego, CA) with a 150‐bp paired‐end model.

### Preparation and Sequencing of 10x Genomics Spatial Transcriptome Samples

Fresh GC tissue samples were collected from the operating room and processed into formalin‐fixed paraffin‐embedded (FFPE) sections (5 µm thickness). Tissue quality was assessed using the DV200 (percentage of RNA fragments > 200 nucleotides), with a minimum threshold of ≥30%. Only high‐quality tissue samples were selected for further processing. The selected tissue sections were placed on Superfrost Plus Microscope Slides (Fisherbrand), followed by H&E staining after deparaffinization. Then, sections were imaged and decrosslinking according to the protocol (CG000520). The human probe V2 panel, which contains a specific pair of probes for each targeted gene, was applied to the deparaffinized and stained tissues and incubated at 50 °C overnight. After probe hybridization, post‐hybridization washes were performed, and ligation buffer was added to repair the junctions between probe pairs that had hybridized to the target RNA. Next, a probe release mix containing RNase and a tissue removal enzyme was added to release the single‐stranded ligation products from the tissue. The released probes were then captured on the Visium CytAssist Spatial Gene Expression Slides within the Visium CytAssist instrument (CG000495) in Berry Genomics (China). The captured probes were extended with UMIs, spatial barcodes, and partial reads. To collect the extended probes, 0.08 M KOH was added, and pre‐amplification was performed for ten cycles. The pre‐amplified products were then purified using 1.2X SPRI select beads. Following the determination of the optimal PCR cycle number for gene expression libraries, the pre‐amplified products were amplified with index primers to ensure no sample index overlap during multiplexed sequencing. After library construction, sequencing was performed using the Illumina NovaSeq 6000 platform in 150 PE mode.

### Multiplex Immunofluorescence Staining

To identify the expression of AREG‐ERBB2 axis in the interaction of iCAFs and tumor cells in the normal, metaplasia, and GC tissues, AREG, ERBB2, SOX9, PanCK and DAPI were used in the multiplex immunofluorescence staining assays with PANO 7‐plex IHC kit (0 004 100100, Panovue). Different primary antibodies were sequentially applied, followed by incubation with horseradish peroxidase‐conjugated secondary antibodies and tyramide signal amplification (TSA). The slides were subjected to microwave heat treatment after each TSA operation. Nuclei were stained with 4‐6‐diamidino‐2‐phenylindole (DAPI) after all human antigens had been labeled. To obtain multispectral images, the stained slides were scanned using the Mantra System (PerkinElmer), which captured the fluorescent spectra at 20‐nm wavelength intervals from 420 to 720 nm with consistent exposure time, the scans were merged to build a single stack image. The extracted images were further used to establish a spectral library for multispectral unmixing by InForm image analysis software (PerkinElmer). Using this spectral library, reconstructed images of sections were obtained with the auto‐fluorescence removed. The antibodies are listed in Table S8 (Supporting Information).

### Isolation of iCAFs from Gastric Cancer Tissues

Isolation and expansion of inflammatory cancer‐associated fibroblasts (iCAFs) from the patient of GC were performed with previously established protocol.^[^ ^37^ ^]^ Briefly, the enzymatically dissociated cells were grown in the culture dishes with alpha modification of Minimum Essential Medium (α‐MEM) containing 10% selected fetal bovine serum (FBS) and 1 ng mL^−1^ basic fibroblast growth factor (bFGF, R&D Systems, Minneapolis, MN), 100 µg mL^−1^ penicillin, and 100 U mL^−1^ streptomycin (Invitrogen, NY, USA). After 48 h, non‐adherent cells and tissue debris were removed and washed twice with phosphate‐buffered saline (PBS). Adherent fibroblasts were further incubated for 6–10 days until 80–90% confluence. Then the mixed fibroblasts were sorted by FACS using BB515 mouse anti‐human CD140α and APC mouse anti‐human CD10 (Cat:17‐0106‐42, Thermo, 1:100) to enrich iCAF subpopulations. The iCAFs were further expanded in the above medium (α‐MEM +10% selected FBS+1 ng mL^−1^ bFGF) and the passage 5–10 iCAFs were used for subsequent experiments in this study.

### Cell Culture

The human GC cell lines AGS and GES‐1 were purchased from Beijing Beina Chuanglian Biotechnology Institute. 293T cells were obtained from American Type Culture Collection (ATCC) and were grown in high glucose DMEM medium supplemented with 10% FBS (Invitrogen). AGS and GES‐1 cell lines were grown in RPMI1640 medium supplemented with 10% FBS (Invitrogen). All cell lines were cultured in a humidified incubator containing 5% CO_2_ at 37 °C.

### Tumor Sphere Formation

A total of 2 000 GC cells were seeded in the 6‐well ultra‐low attachment plates (Corning, USA) and cultured in the sphere medium: DMEM/F‐12 supplemented with B27 (Life Technologies, USA, 1:50), N2 (Life Technologies, 1:100), 20 ng mL^−1^ epidermal growth factor (EGF), 10 ng mL^−1^ bFGF, 100 units/mL penicillin, and 100 ng mL^−1^ streptomycin. GC cells were treated with 10 µM ErbB2 inhibitor, Lapatinib (S2111, Selleck) or DMSO as control. Tumor spheres were counted under stereomicroscope after 7–9 days. In the anti‐tumor drug efficacy analysis, carboplatin (CBP, 10 µg mL^−1^) and Cisplatin (DDP, 1 µg mL^−1^) were added in GC cell sphere formation assays.

### Tumor Sphere Formation in the iCAFs/Gastric Cancer Cell Transwell Co‐Culture System

To assess the effect of iCAFs on the sphere formation of GC cells via transwell co‐culture system, confluent iCAFs were treated with mitomycin C for 1.5 h. Then 5 × 10^5^ iCAFs were placed in the upper inserts of a transwell system. After 24 h of culture, the inserts were transferred into fresh wells of ultra‐low attachment plates supplemented with tumor sphere culture medium. GC cells (2 000 cells) were then seeded in the bottom chamber of 6‐well ultra‐low attachment plate. Tumor spheres were counted under stereomicroscope after 7–9 days. For the anti‐tumor drug efficacy assays, carboplatin (CBP, 10 µg mL^−1^) and Cisplatin (DDP, 1 µg mL^−1^) were added in the iCAFs/GC cell transwell co‐culture system.

### Gastric Tumor Organoid Culture

Freshly processed human gastric tumor tissues were collected from GC patients who underwent gastrectomy at the Department of General Surgery, The First Medical Center, PLA General Hospital (Beijing, China). The application of these samples was conducted in accordance with the Declaration of Helsinki, and the study protocol was approved by Beijing University of Technology Research Ethics Committee. The GC sample was cut finely and washed three times with cold PBS. The tissue debris was transferred to a 50 mL centrifuge tube, including collagenase solution (advanced DMEM/F12 medium, containing 1 mg mL^−1^ collagenase IV and 1x P/S), and incubated for 40 min at 37 °C whiles being gently rotated on a rotator. The tissues were released by pipetting up and down ten times in advanced DMEM/F12, and washed twice with PBS, followed by centrifugation, and resuspension in Matrigel. A 50 µL mixture of the tissue suspension and Matrigel was added to each well of a 24‐well plate. Once the drops solidified, 500 µL of gastric organoid culture medium (advanced DMEM/F12 containing 2 mM GlutaMax, 1x HEPES, 1x P/S, 1x B27, 1x N2, 10% R‐spondin‐1, 10% Noggin, 50 ng mL^−1^ EGF, 200 ng mL^−1^ FGF10, 50 ng mL^−1^ HGF, 1 mM N‐Acetylcysteine, 1 nM Gastrin, 10 mM Nicotinamide, 1 µM Forskolin, 2 µM A83‐01, 10 µM Y‐27632, 20 µg mL^−1^ Dexamethasone, and 100 µg mL^−1^ Primocin) was added to each well to support organoid growth, and organoids were maintained in a 37 °C humidified atmosphere under 5% CO2. Gastric tumor organoids typically appeared after 5–7 days and were passaged every 7–10 days to maintain their growth and viability.

### Short Hairpin RNA (shRNA) and Lentivirus Package

To generate the lentivirus plasmid for stable RNA interference, short hairpins (shRNA) were designed using online software (http://rnaidesigner.lifetechnologies.com/rnaiexpress/design.do). The sequences of the effective shRNA were provided as follows: shAREG: ATTCACGGAGAATGCAAATAT; shERBB2: TGTGGCCTGTGCCCACTATAA; A non‐targeting, scramble silencing RNA was used as control (shCtrl). Lenti‐virus packaging was performed in 293T cells after co‐transfection of packaging plasmids (pRRE, pCMV‐VSVG, and pRSV‐REV, Addgene) using Lipofectamine 2000.

### Quantitative Polymerase Chain Reaction (qPCR)

Total RNAs were purified using TRIzol (Invitrogen). The total RNA was reverse‐transcribed into cDNA with the HiScript III All‐in‐one RT SuperMix Perfect for qPCR kit (Vazyme, China). Gene expression was assayed using the miScript SYBR‐Green PCR Master Mix on the ABI Prism 7900 system. The level of mRNA expression for each gene was normalized to 18s RNA. The qPCR experiments were conducted according to the Minimum Information for Publication of Quantitative Real‐Time PCR Experiments (MIQE) guidelines. The mRNA primer sequences were listed in Table S9 (Supporting Information).

### Western Blotting

Whole cell protein extracts were homogenized in lysis buffer and protease cocktail inhibitor Ι (Calbiochem, San Diego, CA), resolved by SDS‐polyacrylamide gels and then transferred to PVDF membranes. The blots were blocked in TBST containing 5% skimmed milk and incubated with primary antibodies overnight at 4 °C. The membranes were washed with TBST and then incubated with horseradish peroxidase‐conjugated secondary antibodies (Beijing Zhongshan Biotechnology, China) for 1 h at room temperature. After three additional washes with TBST, the antigen‐antibody reaction was visualized by enhanced chemiluminescence assay (ECL, Thermo). The antibodies were listed in Table S8 (Supporting Information). Small molecular inhibitors and growth factors were in Table S10 (Supporting Information).

### Data Processing for scRNA‐Seq

The raw sequencing data were aligned to the GRH38 reference genome using Cell Ranger (10X Genomics, v5) count and vdj function. The count matrices of gene expression from each sample were imported to Seurat.^[^ ^50^ ^]^ High quality cells were selected for further analysis following three criteria: 1)) cells had over than 2 000 UMIs, fewer than 6 000, and more than 300 expressed genes, and less than 10% UMIs derived from the mitochondrial genome; 2) genes expressed in over than ten cells per sample; 3) cell doublets were removed using DoubletFinder^[^ ^51^ ^]^ R package. The remaining high‐quality cells’ expression matrices were integrated with the RunFastMNN function provided by SeuratWrappers R package, followed by normalization to the total cellular UMI count. A union of the top 2 000 genes with the highest dispersion for each data set to generate an integrated matrix. The gene expression matrices were scaled by regressing out the total cellular UMI counts and percentage of mitochondrial gene. The principal component analysis (PCA) was conducted using highly variable genes (HVGs), and the top 30 significant principal components (PCs) were selected to perform UMAP dimension reduction and visualization of gene expression. The harmony R package or the RunFastMNN function derived from the SeuratWrappers R package was employed to remove the batch effects of scRNA‐seq data from multi‐samples. The differentially expressed genes (DEGs) between two groups were identified using the FindAllMarkers function in Seurat, with the min.pct parameter set at 0.2 (i.e., considering only genes expressed in more than 20% of cells). The nonparametric Wilcoxon rank‐sum test was used to obtain the p value for comparisons, the adjusted p value based on Bonferroni correction was calculated. Cell sub‐cluster was annotated based on gene expression pattern and specific marker genes, cell types were assigned in the resulting 2D UMAP using canonical marker genes and the top 30 DEGs.

### Cell Type Abundance Estimation

To characterize the tissue distribution of meta‐clusters, odds ratios (OR) were calculated and used to indicate preferences as previously reported.^[^ ^52^, ^53^ ^]^ Then Fisher's exact test was applied on this contingency table, allowing the calculation of both OR and corresponding p‐value. P‐values were adjusted using the Benjamini‐Hochberg (BH) method implemented in the R function p.adjust. ORs greater than 1.5 or less than 0.5 were found to have adjusted p‐values <1e‐10. An OR greater than 1.5 indicated that meta‐cluster i was more likely to be distributed in tissue j, while an OR less than 0.5 suggested that meta‐cluster i was less likely to be distributed in tissue j.

### Cell‒Cell Communication Analysis at Single Cell Level and Spatial Level

The R package CellChat^[^ ^54^ ^]^ (v2) was utilized to analyze cell‐to‐cell communication among the cellular components within tumor niche. A CellChat object was first established by grouping defined clusters. For analysis, the “CellChatDB.human” ligand‐receptor interaction database was employed for the analysis without additional supplementation, with all preprocessing steps conducted using default parameters. The functions computeCommunProb and computeCommunProbPathway were employed to infer the network of each ligand‒receptor pair and signaling pathway, respectively. Visualization was achieved through a hierarchy plot, circle plot, and heatmap, each serving as a distinct form of visualization.

### Single‐Cell Copy Number Variation Analysis

To classify the malignant epithelium from non‐malignant epithelium, the CNV was analyzed with the R package infercnv^[^ ^55^ ^]^ (v1.8.1), which is designed to infer copy number alterations from tumor single‐cell RNA‐seq data. This package compares expression intensities of genes across malignant cells in advanced and recurrent tumor tissues. Epithelial cells in normal tissues, and the immune cell were used as a reference, as previously reported.^[^ ^18^, ^27^ ^]^

### Data Analysis of Spatial Transcriptomic Sequencing

The raw spatial transcriptomic sequencing reads were checked for quality and mapped using Spaceranger (version 1.2.0). Normalization across spots was performed using the LogVMR function. After quality control using Space Ranger software, the dimensionality reduction and clustering were performed using independent component analysis (PCA) at a resolution of 0.8 with the first 30 PCs. Spatial feature expression plots were generated using the spatial feature plot function in Seurat. To estimate the spatial cell type abundance, the signature genes of tumor cell subsets, CAF subsets, and immune cell subset were obtained from the top 30 marker genes in the corresponding cell type calculated using the FindALLMarkers function in Seurat, and the spatial cell type signature score or the relative abundance was estimated by a deconvolution method, conditional autoregressive‐based deconvolution (CARD).^[^ ^56^ ^]^

### Pathway Enrichment

To assess the cancer single‐cell functional state of epithelium, the GSVA^[^ ^57^ ^]^ package was employed to evaluate the 14 functional states signatures (http://biocc.hrbmu.edu.cn/CancerSEA/). To illustrate the enriched signaling pathways of CAF subtypes, the GSVA^[^ ^57^ ^]^ package was used to assess pathway differences using the C2 curated gene set from the Molecular Signatures Database (MSigDB), with pathway activity calculated using a linear model provided by the limma package.

### Pathway RespOnsive GENes for Activity Inference (PROGENy)

The Pathway RespOnsive GENes for activity inference (PROGENy) package^[^ ^58^ ^]^ was used to infer cancer related pathway activity from the single cell gene expression matrix of tumor epithelium and CAFs as previously reported.^[^ ^58^ ^]^ The results were visualized using a heatmap.

### Trajectory Analysis and Single‐Cell Differentiation States with CytoTRACE

To explore the potential differentiation trajectories between epithelial cells and CAFs subtypes, the trajectory analysis was performed via Slingshot^[^ ^59^ ^]^ and monocle^[^ ^60^ ^]^ package as previously reported.^[^ ^61^ ^]^ For monocle trajectory inference analysis, the monocle object was constructed using “newCellDataSet” function. The DEGs calculated using the “differentialGeneTest” function were selected for the trajectory analysis. Then, Dimensionality reduction was performed using the DDRTree function, and the resulting trajectories were visualized with the plot_cell_trajectory function. For Slingshot trajectory inference analysis, the Seurat object was imported into the Slingshot and PCA‐based dimension reduction was performed with differentially expressed genes, followed by 2D visualization with UMAP. To predict the potential differentiation states among the chief cell, SPEM, and tumor cell as well as CSC, the CytoTRACE trajectory reconstruction analysis using gene counts and expression was applied, as previously reported.^[^ ^31^ ^]^

### TCGA GC Survival Analysis

The Cancer Genome Atlas Program (TCGA) bulk RNA‐seq data, along with curated clinical data from GC patients, were obtained from UCSC Xena website (https://xena.ucsc.edu/). To evaluate the association between the signatures of immune cell, tumor cell and stromal cell types identified by scRNA‐seq with GC patients’ survival, the GSVA^[^ ^57^ ^]^ was used to calculate the cell‐type‐specific signatures score using the top 30 marker genes of each cell type. The association between the abundance of the annotated cell types and patient survival was further evaluated, and the Cox proportional hazards model was conducted, which included age and tumor stage. Kaplan‐Meier survival curves were plotted using the R package “survminer”.

### Statistics

For statistical analysis, the data were analyzed using GraphPad Prism 8.0. For significant testing, the two‐tailed unpaired Student's t‐test was used. *P* < 0.05 was considered significant (*P < 0.05, **P < 0.01, and ***P < 0.001), and *P* > 0.05 was considered non‐significant (ns).

## Conflict of Interest

The authors declare no conflict of interest.

## Author Contributions

M.Z., Y.W., X.Y., and G.P. were responsible for the study concept, design, interpretation and revising the manuscript. G.Z., X.Z., W.P., and X.C. designed the experiments, collected the biopsies, analyzed the data, and wrote the manuscript. L.W., Y.Y., and Y.Z. performed experiments and analyzed data. C.L., S.S., S.Y., Y.G., M.W., and X.W. collected the biopsies and did verification work. L.Z. evaluated gastric tumor pathology.

## Supporting information



Supporting Information

Supporting Information

## Data Availability

The data that support the findings of this study are available in the supplementary material of this article.
