# Supplementary material for: Dissecting the Spatial and Single‐Cell Transcriptomic Architecture of Cancer Stem Cell Niche Driving Tumor Progression in Gastric Cancer
Source: Adv Sci (Weinh). 2025 Feb 14;12(18):2413019. doi: 10.1002/advs.202413019 (PMC12079437; doi:10.1002/advs.202413019)
Supplement: Supplementary file 1 — Supporting Information [file ADVS-12-2413019-s001.docx]

**Supplementary information**

**Dissecting the spatial and single-cell transcriptomic architecture of cancer stem-like cell niche driving tumor progression in gastric cancer**

Guangyu Zhang^1, #^, Xin Zhang^2, #^, Wenting Pan^3, #^, Xizhao Chen^4, #^, Lingfei Wan^3^, Chunjie Liu^5^, Yuting Yong^3^, Yue Zhao^3^, Shuli Sang^5^, Lihua Zhang^6^, Sheng Yao^7^, Yushu Guo^2^, Mingmei Wang^2^, Xinhui Wang^2^, Guangdun Peng^1, *^, Xinglong Yan^3, *^, Yanchun Wang^5, *^, Min Zhang^4, *^

**Affiliations:**

^1^Guangzhou Institutes of Biomedicine and Health, Chinese Academy of Sciences, Guangzhou, China.

^2^Department of Pharmacy, Medical Supplies Center, Chinese PLA General Hospital, Beijing, China.

^3^Beijing International Science and Technology Cooperation Base for Antiviral Drugs, Beijing Key Laboratory of Environmental and Viral Oncology, College of Chemistry and Life Science, Beijing University of Technology, China.

^4^Department of Nephrology, First Medical Center, Chinese PLA General Hospital, Beijing, China.

^5^State Key Laboratory of Pathogen and Biosecurity, Academy of Military Medical Sciences, Beijing, China.

^6^Department of Pathology, Fourth Medical Center, Chinese PLA General Hospital, Beijing, China.

^7^Department of General Surgery, First Medical Center, Chinese PLA General Hospital, Beijing, China.

**This file includes:**

**Figures S1 to S9**


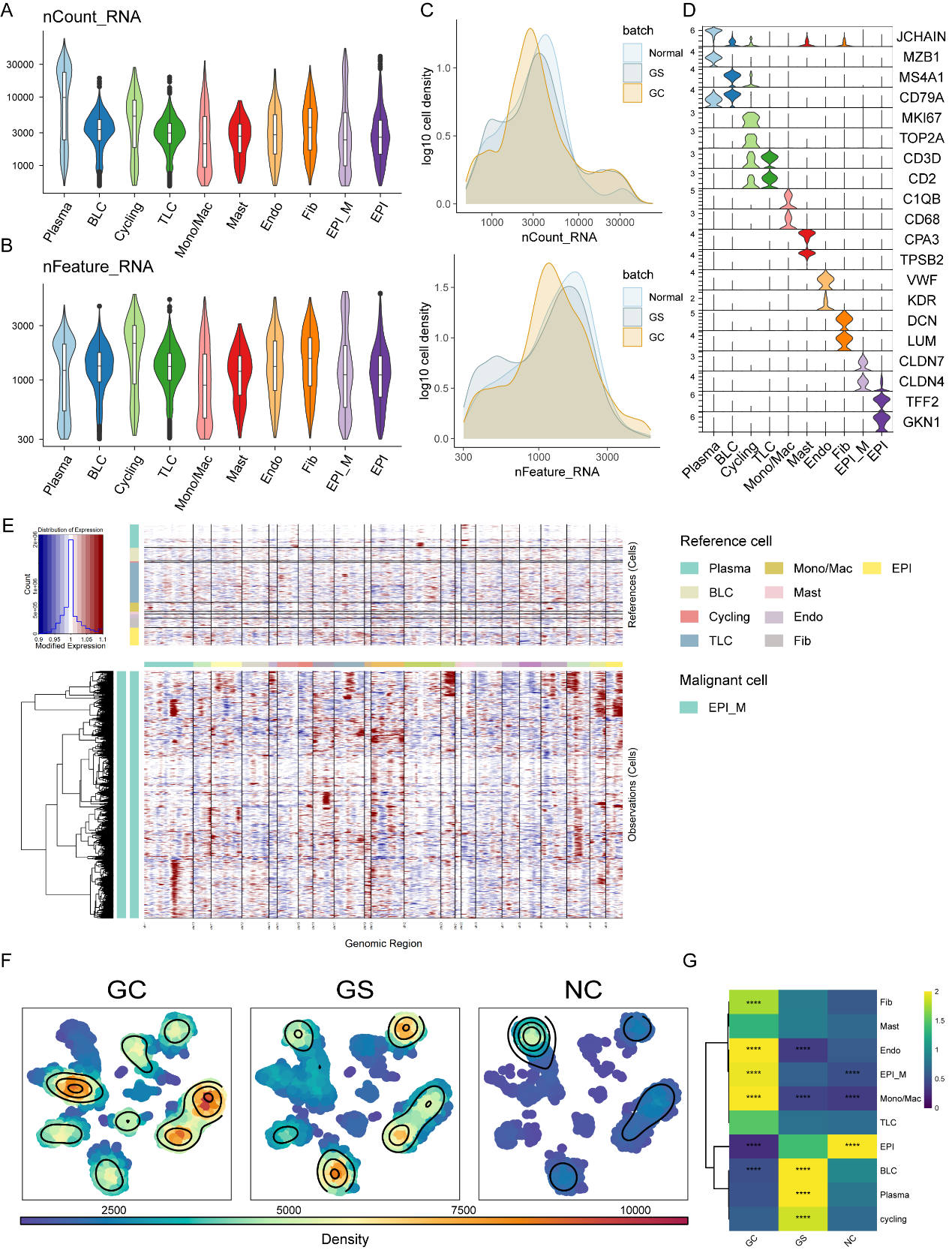


**Figure S1. Single cell RNA-seq data quality control and CNV analysis**

(A-B) The number of detected unique molecular identifier (UMI) counts (A) and genes (B) across all 10 main cell types. (C) The distribution of unique molecular identifier (UMI) counts (upper) and genes (bottom) across three sample groups. (D) The stacked violin plots showing the expression of signature genes across 10 main cell clusters. (E) The heatmap demonstrating the copy number variation (CNV) across different cell types by inferCNV analysis. The column bar, differentiated by colors representing chromosome1-22, accompanies a left-side bar of the heatmap which uses distinct color to indicate different cell types within the gastric tumor microenvironment. The upper section of the heatmap presents the CNV of cell clusters, with non-malignant cells as reference, while the bottom heatmap showing CNV of malignant epithelium. (F) The heatmap depicts the cell distribution density of cell populations across three sample groups. (G) The odds ratios (Ro/e) revealing the cell distribution of 10 main cell types using the STARTRAC-dist index method.


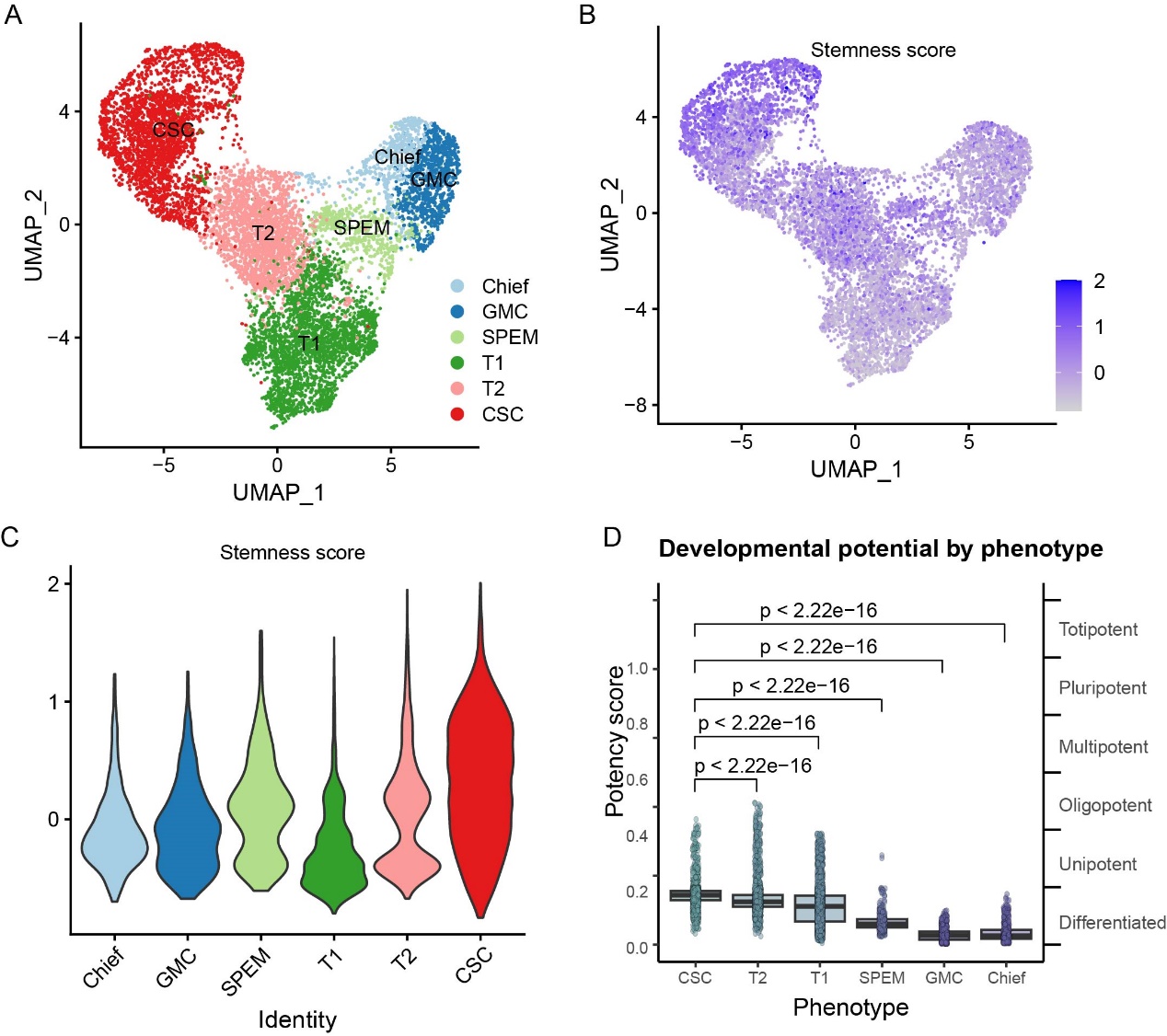


**Figure S2. Molecular characteristic of epithelium during gastric carcinogenesis**. (A) UMAP plot showing epithelial cells subsets distribution. (B) The UMAP showing the stemness signature scores of different epithelium subsets. (C) The violin plot illustrates the stemness signature scores of different epithelium subsets. (D) The boxplot showing the developmental potency score of different epithelium subsets.


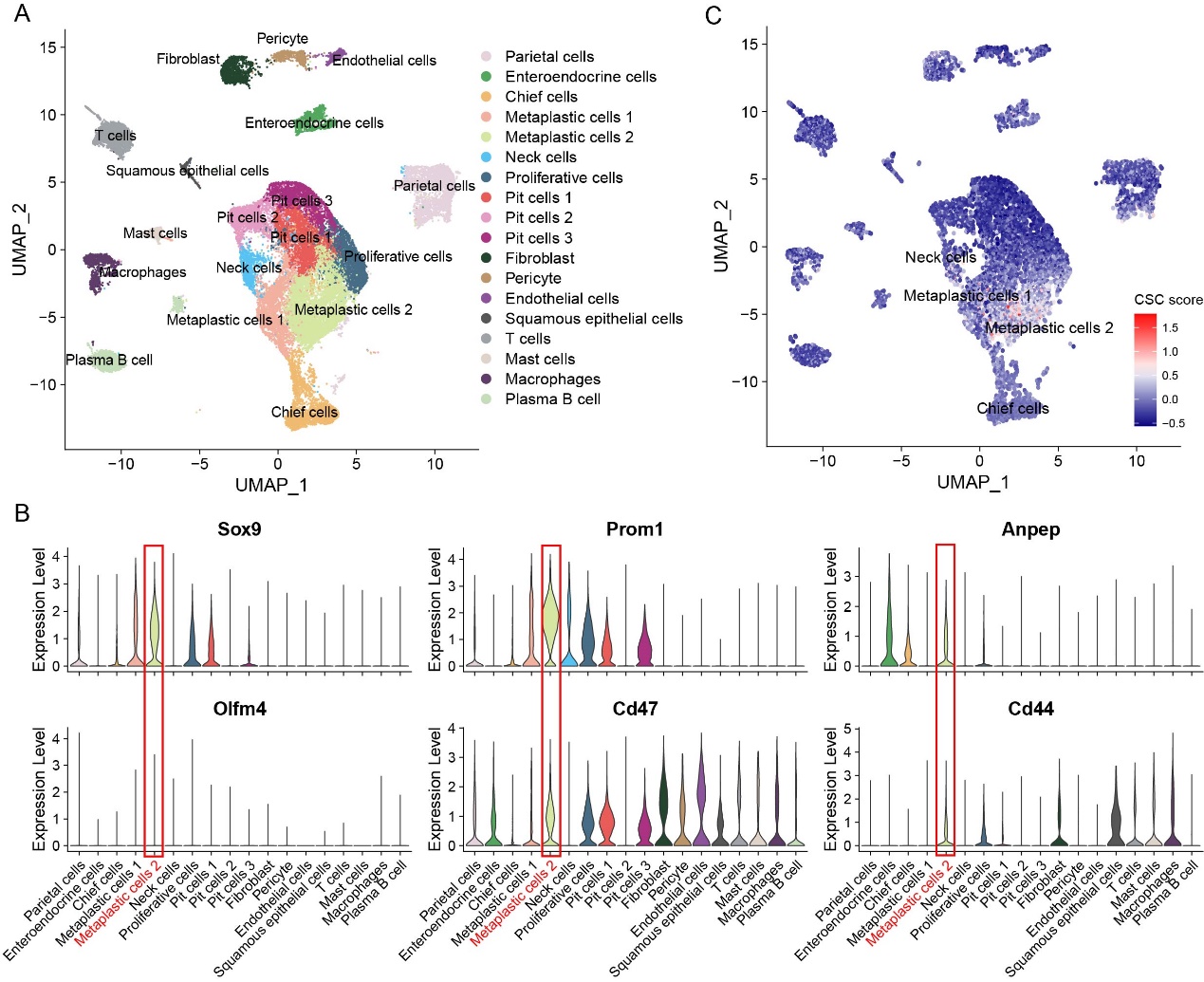


**Figure S3. Re-analysis of single cell transcriptomic data from Hoft et al., 2024 to investigate the molecular characteristics of mouse metaplastic cells**. (A) The UMAP plot showing the cell types distribution in *Helicobactor Pylori* infected and autoimmune induced mouse gastritis. (B) UAMP plot showing the expression of cancer stem cell score (CSC score) in different cell types in *Helicobactor Pylori* and autoimmune induced mouse gastritis. UMAP is colored based on the enrichment of CSC score. (C) The violin plot showing the expression levels of CSC-related marker genes.

**
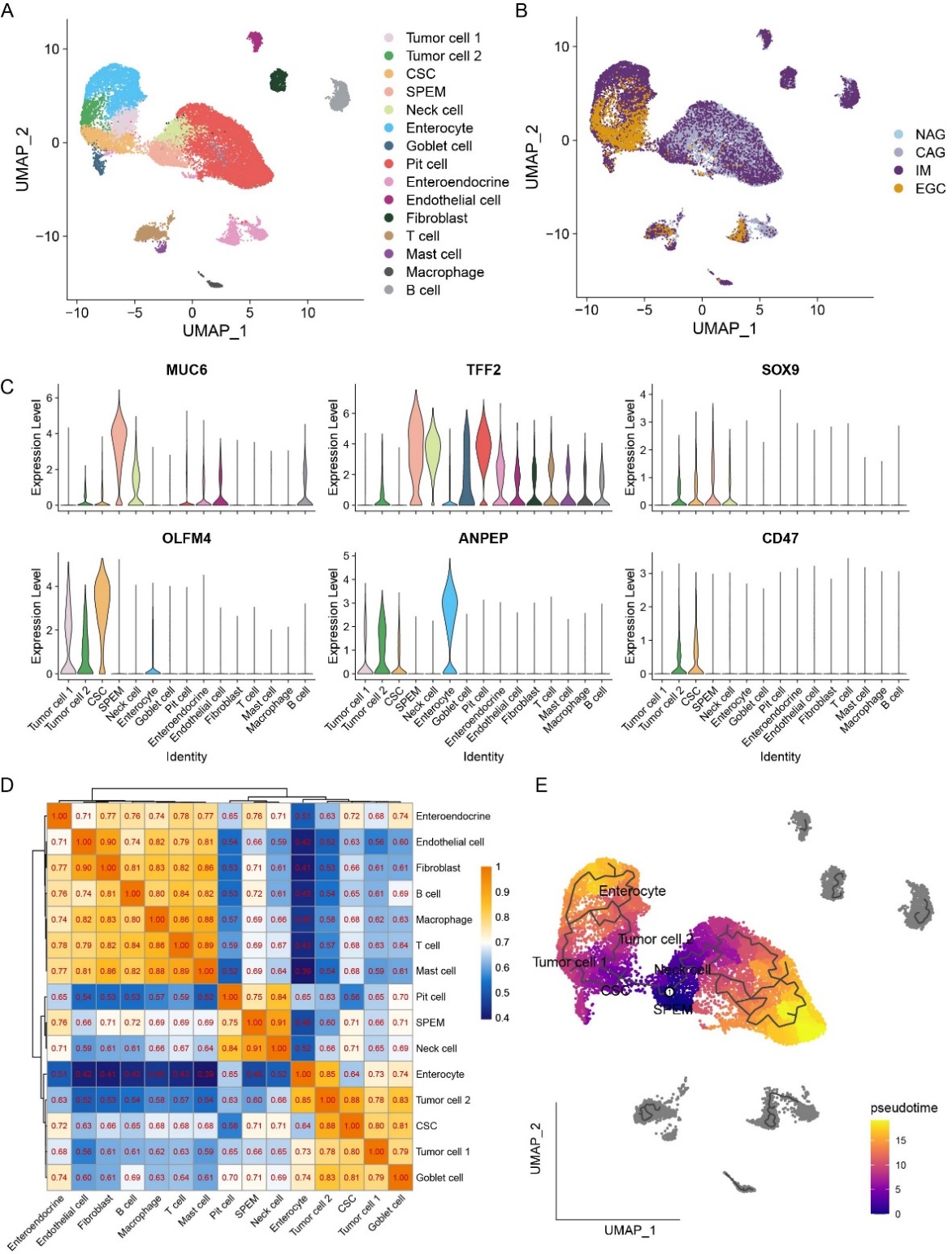
**

**Figure S4. Re-analysis of single cell transcriptomic data from Zhang et al., 2019 to investigate the molecular characteristics of epithelium across different lesions of gastric mucosal samples**. (A-B) The UMAP plot showing the cell types distribution within gastric mucosal samples across non-atrophic gastritis (NAG), chronic atrophic gastritis (CAG), intestinal metaplasia (IM) and early gastric cancer (EGC). UMAP is colored based on the different cell types (A) and different lesions (B). (C) The violin plot showing the expression levels of CSC-related marker genes of different epithelial cell types. (D) The heatmap displaying the similarity of each cell type, colored by the correlation coefficient. (E) The UMAP showing the potential trajectory of all epithelial cell types.


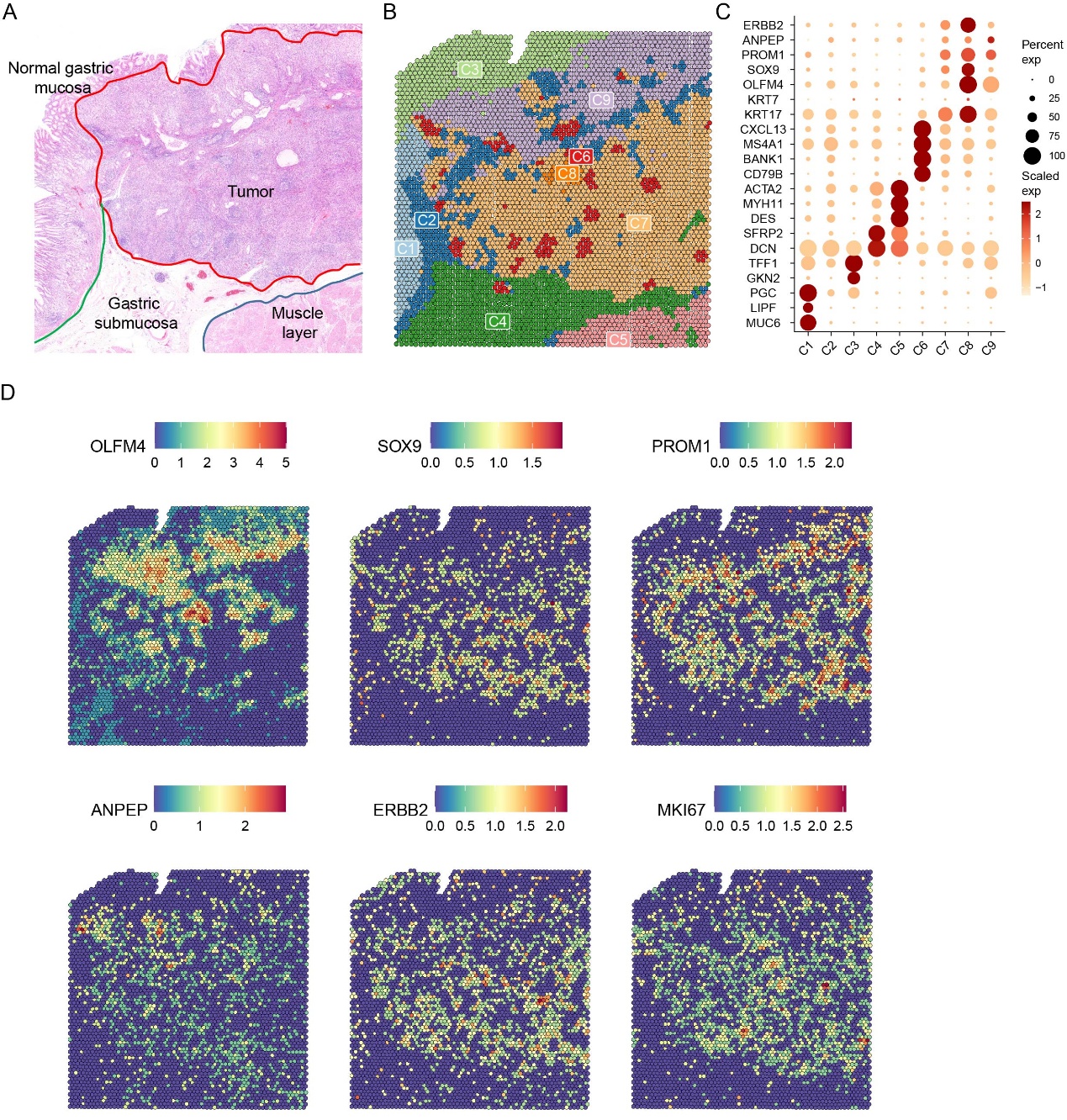


**Figure S5. The spatial transcriptomic characteristics of human advanced gastric cancer.** (A) Representative H&E staining image showing the histology of human advanced gastric cancer. (B) Unsupervised cluster showing the spatial clusters of human advanced gastric cancer. (C) The signature genes of distinct spatial clusters within human advanced gastric cancer. (D) The spatial distribution of CSC related gene expression patterns in situ of human advanced gastric cancer.


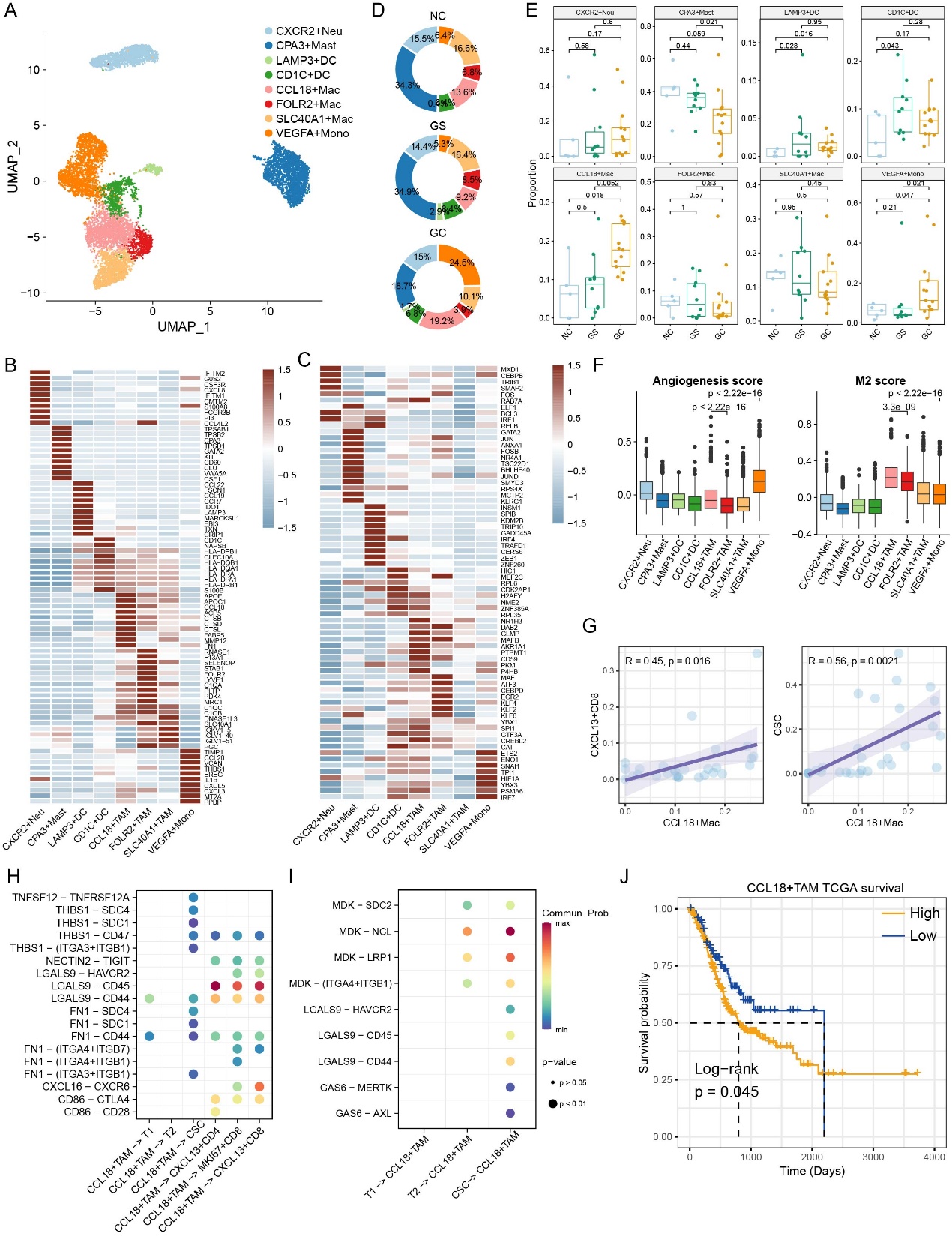


**Figure S6. Characteristics of myeloid subsets during gastric carcinogenesis**. (A)The heatmap depicts eight main subclusters of myeloid cells in gastric tissues with different lesions. (B-C) The heatmap showing the top 10 DEGs (B) and top 10 transcriptional factors (C) in myeloid subclusters. (D-E) The pie chart (D) and box plots (E) revealing the proportion of myeloid subsets in NC, GS, and GC. (F) The boxplot depicts the enrichment of angiogenesis and M2 macrophage related signatures in different myeloid cell subsets, p-values were calculated by student’s t test. (G) The dot plot illustrates the correlation coefficient between CCL18^+^ macrophages with CSCs and CXCL13^+^ CD8 T cells. The oblique line and R represent the average correlation coefficient. P-values were calculated using the Wilcoxon test, and the colors indicate the pathology. (H-I) The bubble plot displaying the ligand-receptor pairs involved in the interactions among different myeloid subsets, T cell subsets, and CSCs. (J) Kaplan-Meier plot illustrating overall survival based on the enrichment of CCL18^+^ macrophages in TCGA GC data, adjusted by age and tumor stage. P-values were calculated by log-rank test.


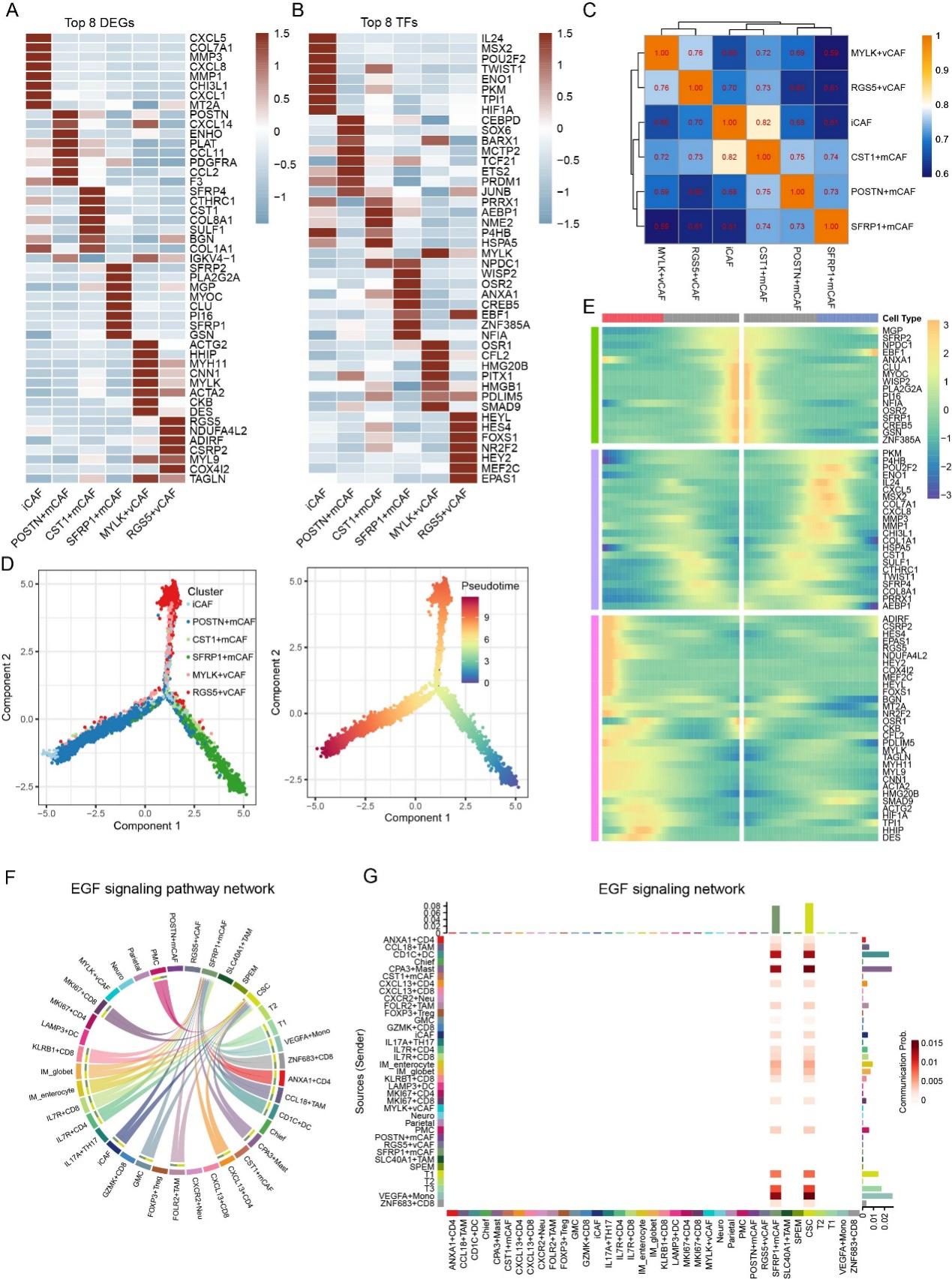


**Figure S7. Characteristics of cancer-associated fibroblast (CAF) subclusters during gastric carcinogenesis.** (A-B) Heatmap showing the top 8 DEGs (A) and top 8 transcriptional factors (B) in CAF subclusters. (C) Heatmap illustrating the transcriptomic similarities of CAF subclusters. (D) Pseudotime analysis, using monocle 2, displays lineage relationship among CAF subclusters. (E) The heatmap showing the continuous change in signature genes of CAF subclusters. (F) The circular plot depicts a representative interacting network through EGF signaling pathways via CellChat analysis. The thickness of the flow represents the relative communication probabilities, while the different color bar indicates different cell types within the gastric TME. (G) The heatmap illustrates the EGF signaling pathway network involved in the interactions between SFRP1^+^mCAFs, iCAFs, and CSCs and their surrounding cellular components. The colors indicate the relative communication probabilities, while the box plots on the top and left panels indicate the proportions of each cell type.


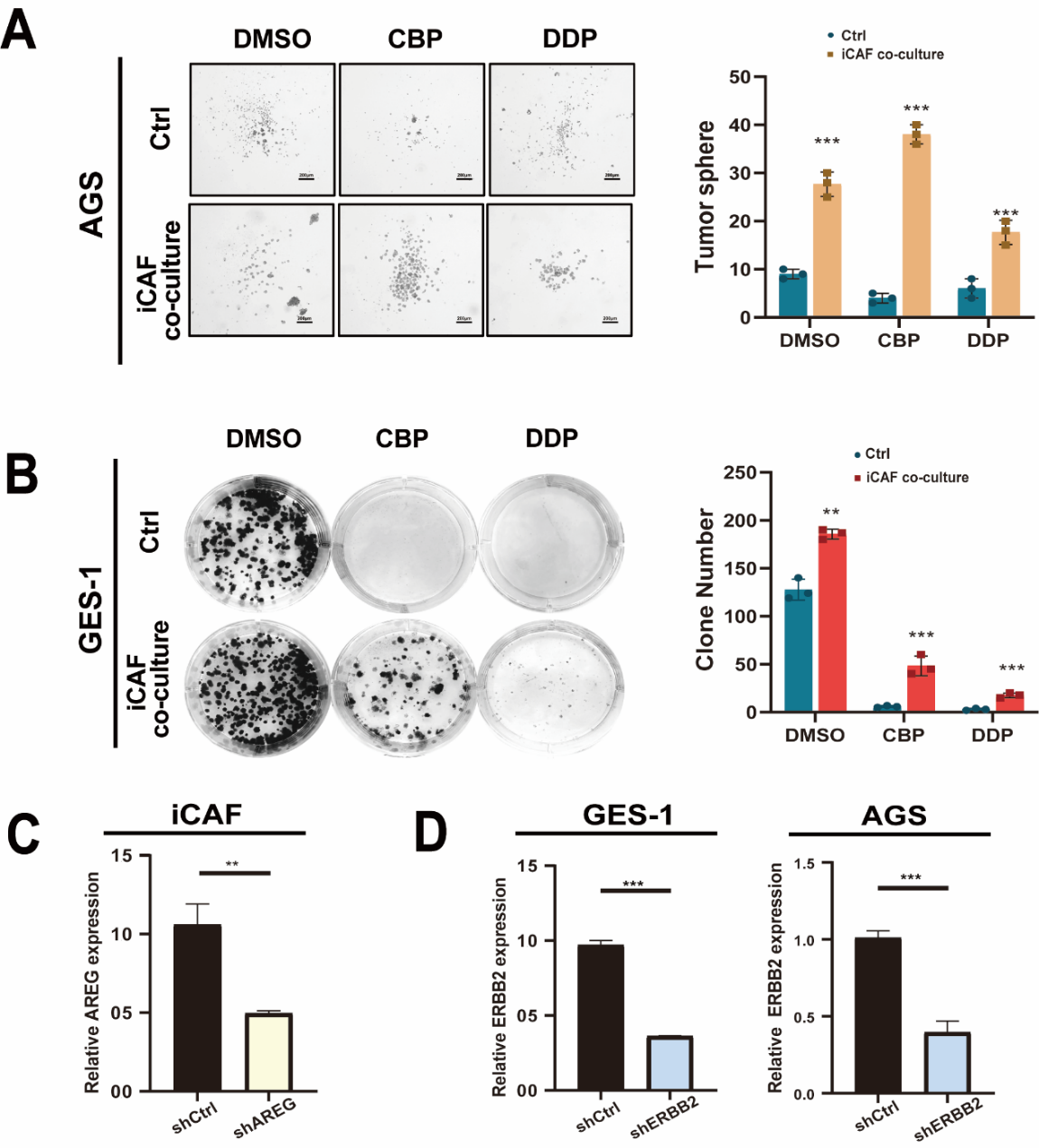


**Figure S8. iCAFs induced chemotherapy resistance of GC cells.** (A) iCAFs induced chemotherapy resistance of AGS cells in tumour sphere formation, with quantitative analysis shown in the right panel. Scale bars: 200μm. (B) iCAFs induced chemotherapy resistance of AGS cells in the clone assays. **P* < 0.05, ***P* < 0.01, ****P* < 0.001 (two-sided unpaired t test). (C) qRT-PCR analysis of lentivirus-mediated RNA interference of AREG in the iCAFs. (D) qRT-PCR analysis of lentivirus-mediated RNA interference of ERBB2 in GES-1 and AGS cells, respectively. **P* < 0.05, ***P* < 0.01, ****P* < 0.001 (two-sided unpaired t test).
